# The eQTL colocalization and transcriptome-wide association study identify potentially causal genes responsible for economic traits in Simmental beef cattle

**DOI:** 10.1186/s40104-023-00876-7

**Published:** 2023-05-11

**Authors:** Wentao Cai, Yapeng Zhang, Tianpeng Chang, Zezhao Wang, Bo Zhu, Yan Chen, Xue Gao, Lingyang Xu, Lupei Zhang, Huijiang Gao, Jiuzhou Song, Junya Li

**Affiliations:** 1 Institute of Animal Science, Chinese Academy of Agricultural Sciences, Beijing, 100193 China; 2 Department of Animal and Avian Science, University of Maryland, College Park, MD 20742 USA

**Keywords:** Cattle, Colocalization, eQTL mapping, GWAS, TWAS

## Abstract

**Background:**

A detailed understanding of genetic variants that affect beef merit helps maximize the efficiency of breeding for improved production merit in beef cattle. To prioritize the putative variants and genes, we ran a comprehensive genome-wide association studies (GWAS) analysis for 21 agronomic traits using imputed whole-genome variants in Simmental beef cattle. Then, we applied expression quantitative trait loci (eQTL) mapping between the genotype variants and transcriptome of three tissues (*longissimus dorsi* muscle, backfat, and liver) in 120 cattle.

**Results:**

We identified 1,580 association signals for 21 beef agronomic traits using GWAS. We then illuminated 854,498 *cis*-eQTLs for 6,017 genes and 46,970 *trans*-eQTLs for 1,903 genes in three tissues and built a synergistic network by integrating transcriptomics with agronomic traits. These *cis*-eQTLs were preferentially close to the transcription start site and enriched in functional regulatory regions. We observed an average of 43.5% improvement in *cis*-eQTL discovery using multi-tissue eQTL mapping. Fine-mapping analysis revealed that 111, 192, and 194 variants were most likely to be causative to regulate gene expression in backfat, liver, and muscle, respectively. The transcriptome-wide association studies identified 722 genes significantly associated with 11 agronomic traits. Via the colocalization and Mendelian randomization analyses, we found that eQTLs of several genes were associated with the GWAS signals of agronomic traits in three tissues, which included genes, such as *NADSYN1*, *NDUFS3*, *LTF* and *KIFC2* in liver, *GRAMD1C, TMTC2* and *ZNF613* in backfat, as well as *TIGAR, NDUFS3* and *L3HYPDH* in muscle that could serve as the candidate genes for economic traits.

**Conclusions:**

The extensive atlas of GWAS, eQTL, fine-mapping, and transcriptome-wide association studies aid in the suggestion of potentially functional variants and genes in cattle agronomic traits and will be an invaluable source for genomics and breeding in beef cattle.

**Supplementary Information:**

The online version contains supplementary material available at 10.1186/s40104-023-00876-7.

## Introduction

Cattle are commonly raised as livestock animals for meat and milk. In the recent decade, thousands of candidate variants and genes for agronomic traits have been detected by genome-wide associated studies (GWAS) in cattle [1]. For example, the variants on *DGAT1* determined the variation of milk production traits [2]. However, the causative variants are still difficult to find due to their tiny effects and many variants in long-range linkage disequilibrium (LD) [3], and how the causative variants contribute to traits and their molecular basis remains largely unknown. Single nucleotide polymorphisms (SNPs) associated with the gene expression are expression quantitative trait loci (eQTLs). The eQTL mapping is more powerful than GWAS in detecting statistically significant genetic effects and revealing inherent biological meaning in the associations between a regulatory variant and its related genes [4–6]. So far, only a few eQTL studies have been conducted in cattle, including milk cells and mammary glands of Holstein cattle [7–9], muscle of Angus-Brahman crossbreed cattle [10], and Nelore cattle [11], and liver and muscle of Irish beef cattle [12]. However, most of these studies only reported the identification and basic characteristics of eQTLs. The comprehensive interpretation of genetic mechanisms of cattle complex traits using efficient multi-omics data strategies, such as transcriptome-wide association studies (TWAS), colocalization between GWAS and eQTLs, and Mendelian randomization (MR), remains rare. Significantly, the eQTLs’ roles in the mechanisms underpinning GWAS linked to agronomic traits in beef cattle remains unclear. Fortunately, as a complement to conventional GWAS, TWAS integrates GWAS and gene expression datasets to identify gene–trait associations [13]. Using sophisticated imputation methodologies [14–16], TWAS has been successful in identifying gene-trait associations and gaining a deeper understanding of the biological mechanisms underlying many complex traits in humans by reducing the burden of multiple testing [17]. MR is a research method that provides evidence for putative causal relations between modifiable risk factors (gene expression) and disease (complex traits) using genetic variants as instrumental variables [18], which has been widely applied to study the relationships among molecular phenotype and complex traits or diseases [19, 20].

Although the cattle Genotype-Tissue Expression (cGTEx) project released highly valuable multi-tissue eQTLs by analyzing publically available RNA-sequencing data [21], the bias of variants detection, population stratification caused by multi-breeds data, and confounding factor from multiple experiment designs need to be addressed. For example, both muscle and adipose are widely distributed tissues in the body of cattle, which could be classified into different types with their distinct characteristics in long-term selection. The muscle fiber types and distributions [22], intramuscular fat contents [23], as well as chemical and fatty acid profiles [24] were various in different muscles. The difference in gene expression patterns between different muscles was considerable [25]. Since the muscle of cGTEx used the blended resources, containing a variety of muscle types from both dairy and beef cattle, which produced the general results. Moreover, the muscle transcriptome shows a noticeable difference between beef and dairy cattle [26], the combined use of dairy and beef data may result in a bias. These eQTLs, specific to a certain muscle type or breed, would be lost. A similar problem can be seen in adipose tissue. Therefore, further research is required to improve the quality of eQTL identification, especially in these primary tissues such as muscle and adipose. Compared to other muscle types, the *longissimus dorsi* muscle is more tender, contains more intramuscular fat, and has better flavor, which is the most valuable part of beef. Backfat thickness is an important economic trait in beef cattle. Backfat thickness can affect both yield [10] and flavor and juiciness of beef [4]. The liver plays a key role in weight gain since it is metabolically active and accounts for approximately 24% of basal energy expenditure [27]. However, the eQTL studies for the importantly specific *longissimus dorsi* muscle, backfat and liver, and their roles in the mechanisms underlying GWAS of agronomic traits in beef cattle, remain unknown.

**Fig. 1:**
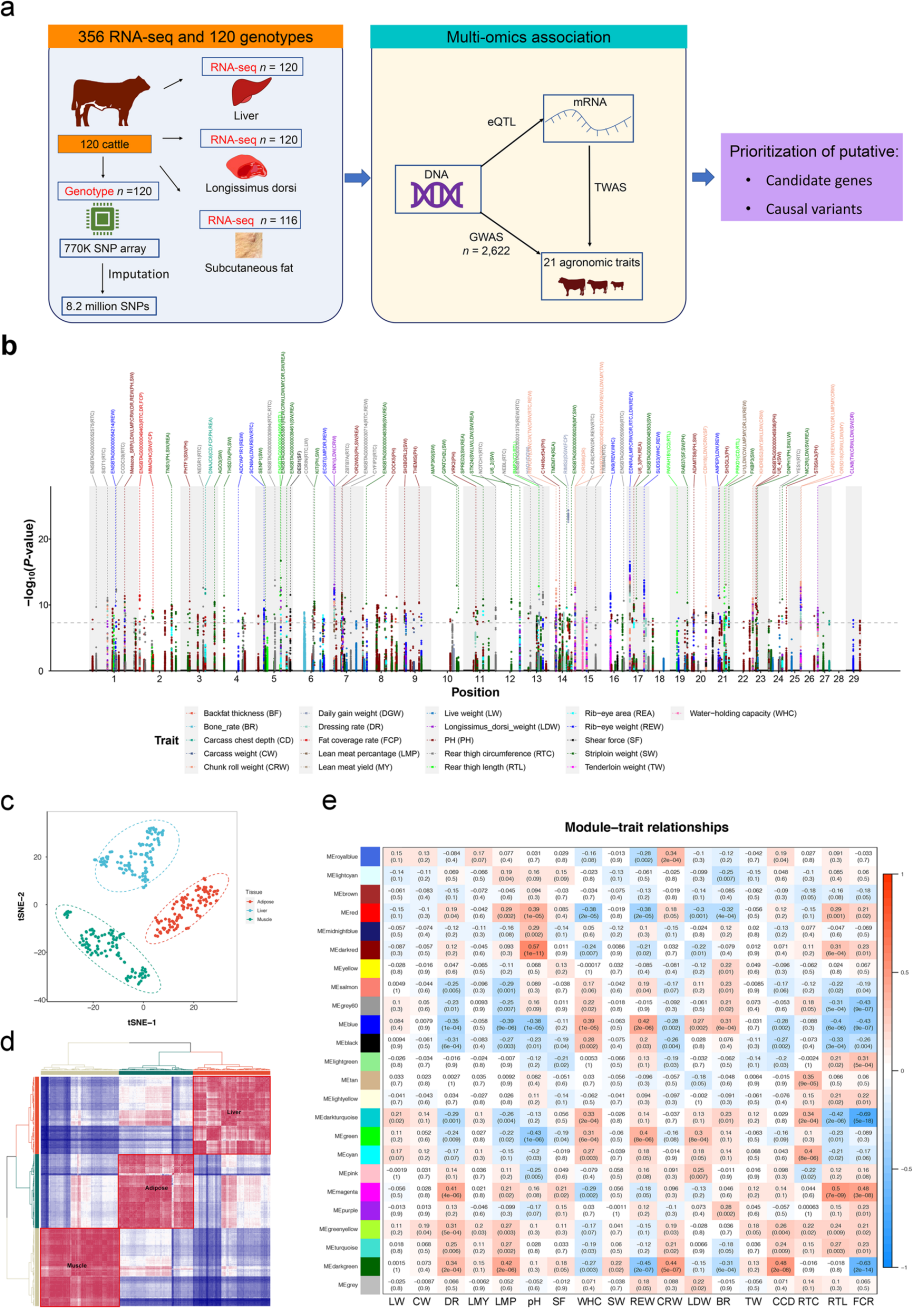
Study design and transcriptome associated with traits. **a** We investigated the molecular characteristics by profiling genotype, and mRNA from liver, muscle and adipose tissue of 120 Simmental cattle with important agronomic traits. We identified the promising candidate genes and causal variants using a multi-omics association strategy. **b** The Manhattan plot of 21 agronomic traits. Only significant variants and their nearby SNPs within up/downstream 100 kb are shown in the plot. The closest gene and associated traits of each sentinel SNP were labeled on the top. **c** Sample clustering using t-SNE based on gene expression levels. **d** Pearson correlation (heatmap) and hierarchical clustering (tree) of transcriptome profiles across 356 samples (rows/columns) show tissue-specific clustering (colors). **e** Correlation of gene co-expression modules with agronomic traits in muscle. Modules were denoted by different colors. Correlation of module eigengene with each agronomic trait displayed in the corresponding box (top: coefficient, bottom: *P*-value). The color of each box represents a positive correlation (red) or a negative correlation (blue)

Simmental cattle is a well-known breed sharing a good reputation in both beef and milk production. Moreover, Simmental cattle is a primary beef breed in China. Therefore, we hypothesized that the GWAS strategy aligning with the aid of the transcriptomic analyses could help decipher candidate genes and causative variants vital to agronomic traits. In this study, we first ran a comprehensive GWAS analysis for 21 agronomic traits using imputed whole-genome variants in Simmental beef cattle. We then applied eQTL mapping in three tissues (*longissimus dorsi* muscle, backfat, and liver) using 356 samples from 120 cattle (Fig. 1a and Fig. S1), followed by a meta-analysis across three tissues to increase our discovery power. We integrated GWAS results with the eQTL of three tissues to prioritize genes and variants affecting agronomic traits via transcriptome. The results from the study present valuable resources on the impact of genetics and propose the underlying genetic architecture of potentially causative variants and candidate genes in cattle agronomic traits.

## Methods

### Animals and experimental design

One Hundred and Twenty healthy Simmental beef cattle born between 2017 and 2018 in Wulagai (Inner Mongolia Autonomous Region in China) were selected for sampling. The yearling steers were delivered to a contract feeder where they were fed a typical feedlot diet consisting of corn, protein, vitamins, and minerals until they reached an average age of 24 months. The 120 blood samples were collected before they were taken to the slaughter room. To ensure consistency of sampling location for each tissue, we collected backfat (subcutaneous adipose) and *longissimus dorsi* muscle between the 12^th^ and 13^th^ rib after slaughtering. Liver tissue was harvested from the edge of the left lobe of the liver. All samples were instantly frozen in liquid nitrogen for total RNA extraction. DNA samples extracted from blood were genotyped using the Illumina BovineHD 770K Beadchip (Illumina Inc., San Diego, CA, USA).

### RNA sequencing and expression profiling

Total RNA was extracted from muscle, liver, and adipose tissue using the Trizol method according to the manufacturer’s instructions. We successfully generated 356 transcriptome libraries from 120 *longissimus dorsi*, 120 liver, and 116 backfat (4 failures). The transcriptome libraries were sequenced using Illumina 150 bp paired-end strategy libraries. Quality trimming and adaptor removal of the Illumina reads were carried out using Cutadapt v2.8 [28] and Trimmomatic v0.39 [29]. After quality control, the clean reads were mapped to the reference genome (ARS-UCD 1.2) using HISAT2 [30]. We used the mapped reads to quantify gene expression using Ensembl 103 annotations. StringTie was used to calculate the per kilobase per million mapped reads (TPM) for each gene among samples [31]. The gene with a threshold of TPM ≥ 0.1 in ≥ 20% of samples was defined as the expressed gene. The gene modules with distinct expression patterns were calculated by weighted correlation network analysis (WGCNA) [32]. All expressed genes were used for module constructions.

### Imputation and GWAS

The phenotype and genotype data of 21 traits were collected from 2,622 individuals over the past decade, including our newly collected data from the above 120 cattle. The summary of phenotype records is shown in Table S1. All 2,622 individuals were genotyped using the Illumina Bovine 770K Bead chip. The SNPs with minor allele frequencies < 0.05, genotype call rates < 90%, located in non-autosome and significant Hardy–Weinberg disequilibrium at 1 × ${10}^{-6}$, as well as samples with call rates < 90% were removed from the downstream analysis using PLINK 1.90 [33]. After quality control, a total of 590,065 variants remained. We then imputed the SNPs to sequence variants level based on a multiple breeds reference panel by Beagle 5.4 [34]. The reference panel consists 1,847 individuals (including 113 Simmental cattle) and was downloaded from https://www.ebi.ac.uk/ena/browser/view/ERZ1738264 [35]. We removed variants with MAF < 0.05 or dosage *R*-squared (DR^2^) < 0.8. After quality control (same as above), 8,221,244 autosomal variants were obtained for GWAS and eQTL mapping. The average DR^2^ of the imputed variants was 0.92. Before performing GWAS, the phenotype was adjusted by year, sex, age, and the first two principal components (PCs) of the genotype generated by PLINK and normalized by rank-transformation using the transform function in GenABEL [36]. We performed an association test for each SNP based on a linear mixed model:

$${\varvec{y}}={\varvec{X}}{\varvec{\beta}}+{\varvec{u}}+{\varvec{\varepsilon}},$$

where ***y*** is adjusted phenotype; ***X*** is a vector of genotypes of a variant at the locus tested; ***β*** is the effect size of the variant; ***u*** is a vector of random polygenic effects ~ ***N*** (0, ${{\varvec{G}}{\varvec{\sigma}}}_{{\varvec{g}}}^{2}$), where ***G*** is genomic relationship matrix constructed from all variants; ***ε*** is a vector of residual errors. Variance component estimation via restricted maximum likelihood (REML) analysis was implemented in GCTA software [37]. We used *P*-value < 5 × 10^−8^ as significance thresholds of GWAS for all traits. We compared GWAS results with Cattle QTLdb (release 47, Apr 25, 2022) [1]. The QTLs that were within ± 100 kb of a QTL/association for the same trait(s) of the Cattle QTLdb were treated as the newly detected QTLs.

### The eQTL mapping

The genes with expression ≥ 0.1 TPM in ≥ 20% of samples were obtained and their values were normalized across samples by the inverse normal transformation. To control for population effects, the first two PCs of genotype were included in eQTL analyses. We estimated latent covariates for gene expression levels for each tissue using the probabilistic estimation of expression residuals (PEER) [38]. We used 5 PEER covariates as confounding variables for gene expression because the posterior variances of factor weights nearly reached plains (Fig. S5). We conducted *cis*-eQTLs mapping using fastQTL [39]. The SNPs located within 1 Mb up/downstream of the transcription start sites (TSSs) were defined as potential *cis*-eQTLs. We applied the nominal *P*-value threshold that corresponds to false discovery rate (FDR) ≤ 0.05 for each gene. To identify independent secondary signals, we treated the most significant corresponding eVariant as a covariate. We repeated *cis*-eQTL mapping until no additional independent signals were detected. The genotype, expression and covariates were used to map *trans*-eQTLs using the MatrixQTL’s linear model [40]. The *P*-values were adjusted by multiple testing using Benjamini–Hochberg (BH) method to obtain FDR. The eQTLs were annotated in genomic regions using the Variant Effect Predictor [41]. Chromatin region data were from a previous study [42]. Enrichment of eQTLs in specific genomic regions was estimated using GAT1.0 with 10,000 permutations [43]. KEGG analysis of gene lists was computed using the GOstat R package with FDR ≤ 0.05 [44]. To evaluate the replication of *cis*-eQTLs in external data sets, we compared the eQTL results with the cattle GTEx project's eQTL summary data, which included 27 tissues and cell types [10]. The replication rate of eQTL across tissues was estimated as the π_1_ statistic using the qvalue R package [45]. The genomic region containing multiple genes regulated by the same eQTL signal was defined as an eQTL cluster. We obtained all significant eVariants regulating at least three genes and merged them into potential eQTL clusters by combining variants located within a distance of < 1 Mb. The cluster containing all eGenes was examined for colocalization of their associated variants using the Coloc package in R [46]. The eGenes-associated variants had the same underlying eQTL signal using a posterior probability (H4) > 0.8 as a cutoff.

### Fine-mapping and multi-tissue eQTL mapping

We conducted fine-mapping of eQTLs for each gene using priors by deterministic approximation of posteriors (DAP) [47], which considers the distance from TSS and LD information (*r*^2^ > 0.25) among SNPs. The priors were estimated by TORUS based on the distances of variants to the TSS of target genes [48]. Any variant with posterior inclusion probabilities (PIP) > 0.8 was considered as fine-mapped result. The meta-analysis was performed in all three tissues for all variant-gene pairs that were significant in at least one of the three tissues based on the eQTL results of single-tissue. We used METASOFT to calculate a posterior probability for each variant-gene pair and tissue tested [49], which is the probability that an eQTL effect exists in a given tissue (called m-value), given the profile of eQTL effect sizes and standard errors across all three tissues. We used an m-value ≥ 0.9 as a threshold to select high-confidence eQTLs.

### TWAS analysis

The genomic relationship matrix was constructed using SNPs within 1 Mb upstream and downstream of each gene's TSS. We estimated the *cis*-SNP heritability (*cis*-${h}^{2}$) for each gene using the REML algorithm in GCTA [37]. The covariates were the same as in the eQTL mapping analysis. The estimation of *cis*-${h}^{2}$ of genes significantly different from zero (by likelihood ratio test) after BH correction with FDR < 0.1 were defined as *cis*-heritable genes. TWAS analysis was limited to these *cis*-heritable genes. The estimation of cis-variant effects on gene expression was based on the expression values of three tissues and the corresponding imputed genotypes in 120 individuals using GCTA [37]. We then predicted the gene expression values for our existing GWAS population of 2,622 cattle using PLINK [33]. The associations between the predicted expression values and the carcass traits were calculated using linear models. The significant associations with defined using FDR ≤ 0.05 as a cutoff.

### Colocalization of eQTL and GWAS

Colocalization between GWAS signals and eQTLs was performed using the Coloc R package [46]. All variants within the 100 kb flanking regions around the index variants were tested for colocalization. GWAS summary statistics were from 21 growth, carcass and beef quality traits in this study, and 36 milk production, reproduction, and body conformation traits published in a previous study [50]. We considered GWAS variant and eVariant pairs as colocalized using the threshold of H4 > 0.8. We performed a Mendelian randomization-equivalent analysis based on the summary statistics of GWAS and eQTL studies using summary data-based Mendelian randomization (SMR) software [51]. We investigated the associations between gene expression (exposure) and a target phenotype (outcome), using the top-associated eQTL in the *cis* region as an instrumental variable. We obtained significant results using a BH-corrected *P*-value threshold (FDR ≤ 0.05). Circos software was used to plot the multi-omics results [52].

## Results

### GWAS for 21 agronomic traits

We analyzed the GWAS between imputed whole-genome variants and 21 agronomic traits using a mixed model approach (Table S1). Using a cutoff of *P < *5 $\times {10}^{-8}$, we found that 1,580 unique variants were associated with the 21 agronomic traits (Fig. 1b). The number of significant variants for each trait ranged from three for the backfat depth trait to 647 for the meat pH trait (Table S1). These significant signals could be clumped into 265 QTL regions, of which 53 QTL regions were associated with at least two traits with the same sentinel SNPs, showing clear genetic pleiotropy effects (Table S1). For example, the sentinel SNP 25:40,477,045 nearby *CARD11* (caspase recruitment domain family member 11) was associated with six carcass traits. We observed that 1,372 associations were newly identified in our analysis, while 208 associations were previously reported in the cattle QTLdb [1].

### Transcriptome profile of three primary tissues

We acquired 16.22 billion clean reads from 356 RNA-seq samples containing 120 *longissimus dorsi*, 120 liver, and 116 adipose tissue. Approximately 96.96% of the total reads were mapped to the reference genome (Table S2 and Fig. S2). Under the expression threshold of TPM ≥ 0.1 in ≥ 20% of samples, 18,789 (68.1%), 17,775 (64.4%), and 16,869 (61.1%) genes were expressed in muscle, liver, and adipose tissues, respectively. Using the t-Distributed Stochastic Neighbor Embedding (t-SNE) algorithm, we separated samples from different tissues and recapitulated the relationships between tissues based on expression levels (Fig. 1c). Hierarchical clustering showed that the expression profiles accurately reflected the tissue type (Fig. 1d and Fig. S3). When we estimated the variance explained by the two PCs of genotype and five confounding factors per gene (Methods, Fig. S4), we discovered that residuals explained most of the variance per gene (mean = 50%, Fig. S5). We detected that 18 traits were correlated with gene modules using WGCNA. We found 75, 99, and 72 significant module-trait pairs for adipose, liver, and muscle, respectively (FDR < 0.05; Fig. 1e, Fig. S6 and Table S3). Functional enrichment analysis revealed that various metabolic pathways coexisted within trait-related modules (Table S3). For example, glutathione metabolism, PPAR signaling pathway, and fatty acid degradation were enriched in the blue module of muscle, which was significantly correlated with several beef production and quality traits, such as dressing rate, lean meat rate, pH, and water-hold capability.

### Genetic regulatory effects on gene expression in three tissues

**Fig. 2:**
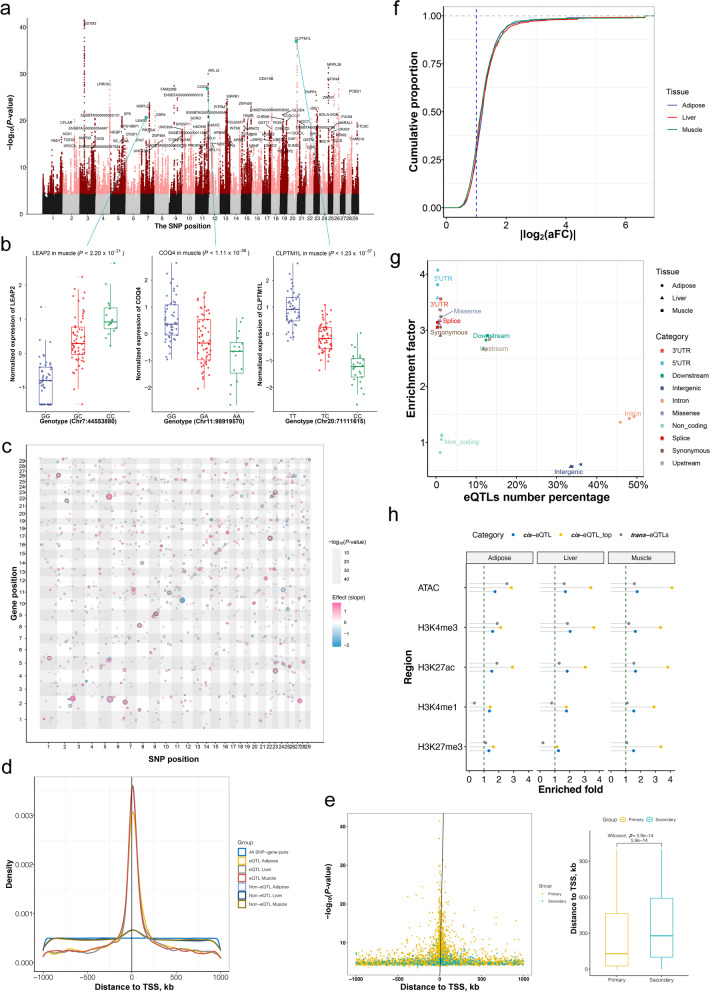
eQTLs in three tissues. **a** Manhattan plot showing the nominal *P*-value (*y*-axis) for all *cis*-eQTLs in muscle. **b** The TPM normalized expression of *LEAP2* with three genotypes. **c** Dot plot showing the locations, *P*-value, and effect sizes for all significant *trans*-eQTL in muscle. Variants and gene positions are shown on the *x*-axis and *y*-axis, respectively. Each dot was a significant *trans*-eQTLs (FDR < 0.05). The size of each dot represents the −log10 scaled *P*-values. The color of each dot represents the direction of the slope effect. **d** Distribution of *cis*-eQTLs around TSS. All SNP-gene pairs indicate all tested SNP-gene pairs. Non-eQTL indicates the top associated SNP for non-eGenes. **e** The distance of the most significant eVariant to the TSS of eGene in muscle. The primary signals (golden) and the secondary signals (blue) relative to TSS are shown using a point plot (left) and their absolute distances compare shown in the box plot (right). The Wilcoxon test is used to compute significance. **f** The absolute allelic fold change distribution for *cis*-eQTLs in three tissues. **g** The proportion and enrichment of *cis*-eQTLs in genome location. The enrichment factors are based on the number of *cis*-eQTLs in each region category divided by the expected number. **h** Enrichment of eQTLs in five chromatin states predicted from a tissue-matched cattle dataset. The *x*-axis represents the enriched fold

**Fig. 3:**
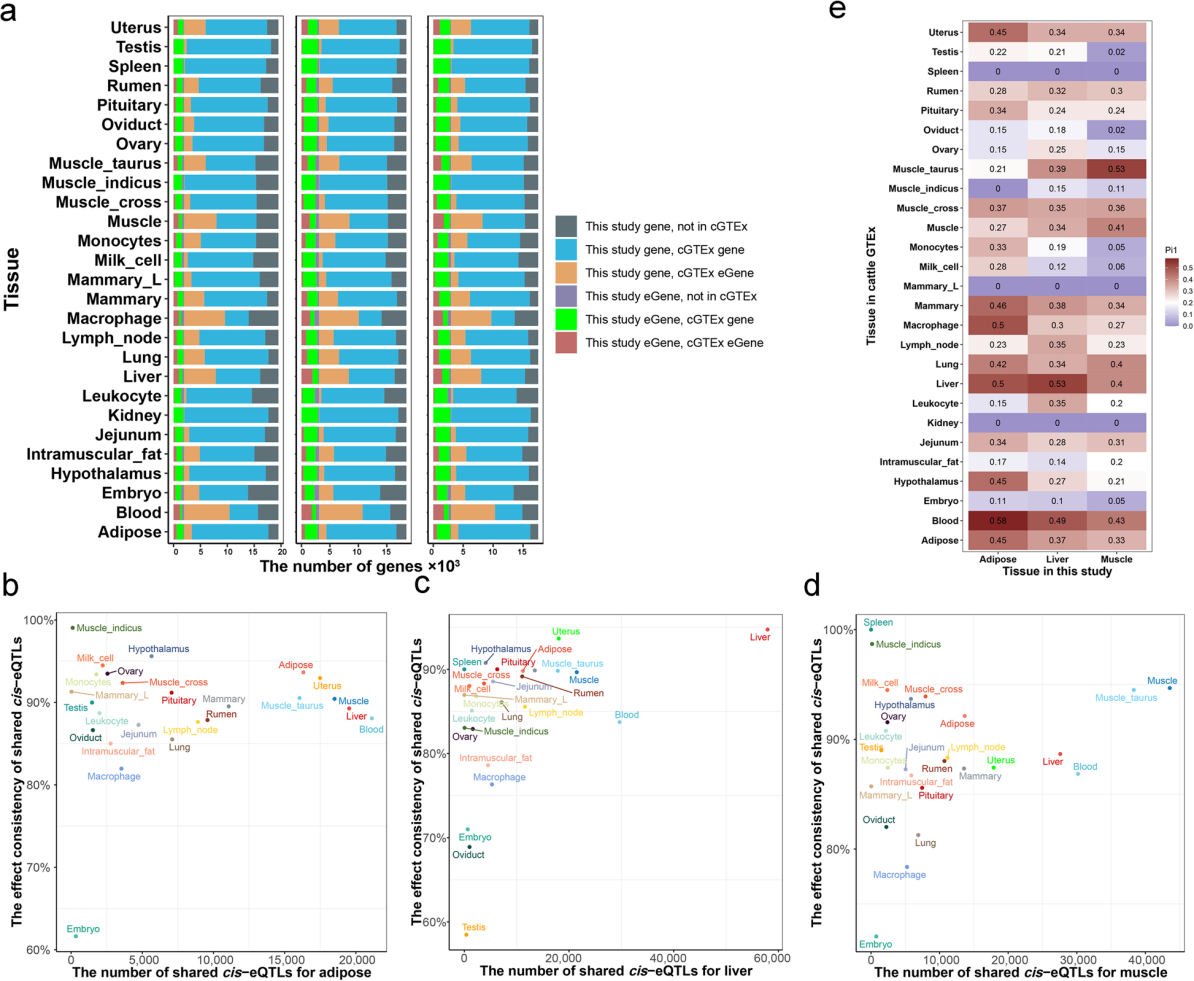
*Cis*-eQTL replication in cGTEx. **a** The overlapped eGene between this study and cGETx for adipose, liver and muscle tissue. **b–d** The allelic directions in adipose, liver, and muscle were highly consistent with the matched tissue of cGTEx. **e** Pairwise sharing patterns (π_1_ value) of *cis*-eQTL between three tissues of this study and 27 tissues/cell types of cGTEx

Considering all three tissues, we identified 854,498 *cis*-eQTLs for 6,017 genes, accounting for 29.1% of all autosomally expressed genes (Fig. S7). The number of eGene (gene with significant *cis*-eQTLs) was 1,952, 3,091, and 2,950 for adipose, liver, and muscle, respectively (Fig. 2a and Fig. S8). A total of 517,478 genetic variants regulated genes in at least one tissue (eVariants, Fig. S7). These eQTLs exhibited significant genotype-driven differences in gene expression among individuals and frequently affected biologically necessary gene expression levels in the respective tissues, such as the body weight and growth function genes *LEAP2* (liver enriched antimicrobial peptide 2), *CPLTM1L* (CLPTM1 Like) and *COQ4* (coenzyme Q4) in muscle (Fig. 2b). A total of 46,970 *trans*-eQTLs for 1,903 genes were detected in the three tissues, including 627, 659 and 789 eGenes in adipose, liver, and muscle, respectively (Fig. 2c and Fig. S7 and S9). The majority of *cis*-eQTLs were centered on TSSs of the target genes (Fig. 2d). Closer to TSSs, we discovered an enrichment of low *P*-values (Fig. S10). We found that 368, 402, and 139 eGenes have at least two independent *cis*-eQTLs in liver, muscle, and adipose, respectively (Fig. S11a). Notably, the most significant eVariants of the primary analysis were significantly located closer to the TSSs when compared with eVariants identified by the conditional analysis (Wilcoxon test, *P*-value < 5.9e−14, Fig. 2e and Fig. S11b, c). Using allelic fold change (aFC), an average of 36% of *cis*-eQTLs had a greater than twofold impact on gene expression across tissues (Fig. 2f). Estimates of heritability revealed that the eGene had higher heritability than non-eGene (Wilcoxon test, *P*-value < 2.2e−16, Fig. S12a). The gene with high heritability also had a more significant *P*-value for *cis*-eQTL mapping (the average Pearson correlation was 0.78, Fig. S12b–d). Although 49.3% eVariants were found in intronic regions, we detected a strong enrichment for 5'UTR and 3'UTR, while intergenic regions were underrepresented when compared to all SNPs evaluated in the dataset (Fig. 2g). The eQTLs were enriched in functional elements of the genome (Fig. 2h), especially for the top eVariant of eGene (Fig. S12e). These *cis*-eGenes were more likely to be expressed at high levels (Fig. S12f). We observed that 29.3%, 59.8%, and 62.9% eGenes of backfat, longissimus dorsi muscle, and liver were replicated in adipose, muscle, and liver of cGTEx, respectively (Fig. 3a). An average of 94.3% concordance in allelic directions among the *cis*-eQTLs effect replicated in the matched three tissues of cGTEx data (Fig. 3b-d). The replication was quantified using the π_1_ statistic, with relatively high replication rates for all three tissues compared with other tissues (Fig. 3e).

### Tissue pattern and pleiotropic of eQTLs, and multi-tissue eQTL Mapping

**Fig. 4:**
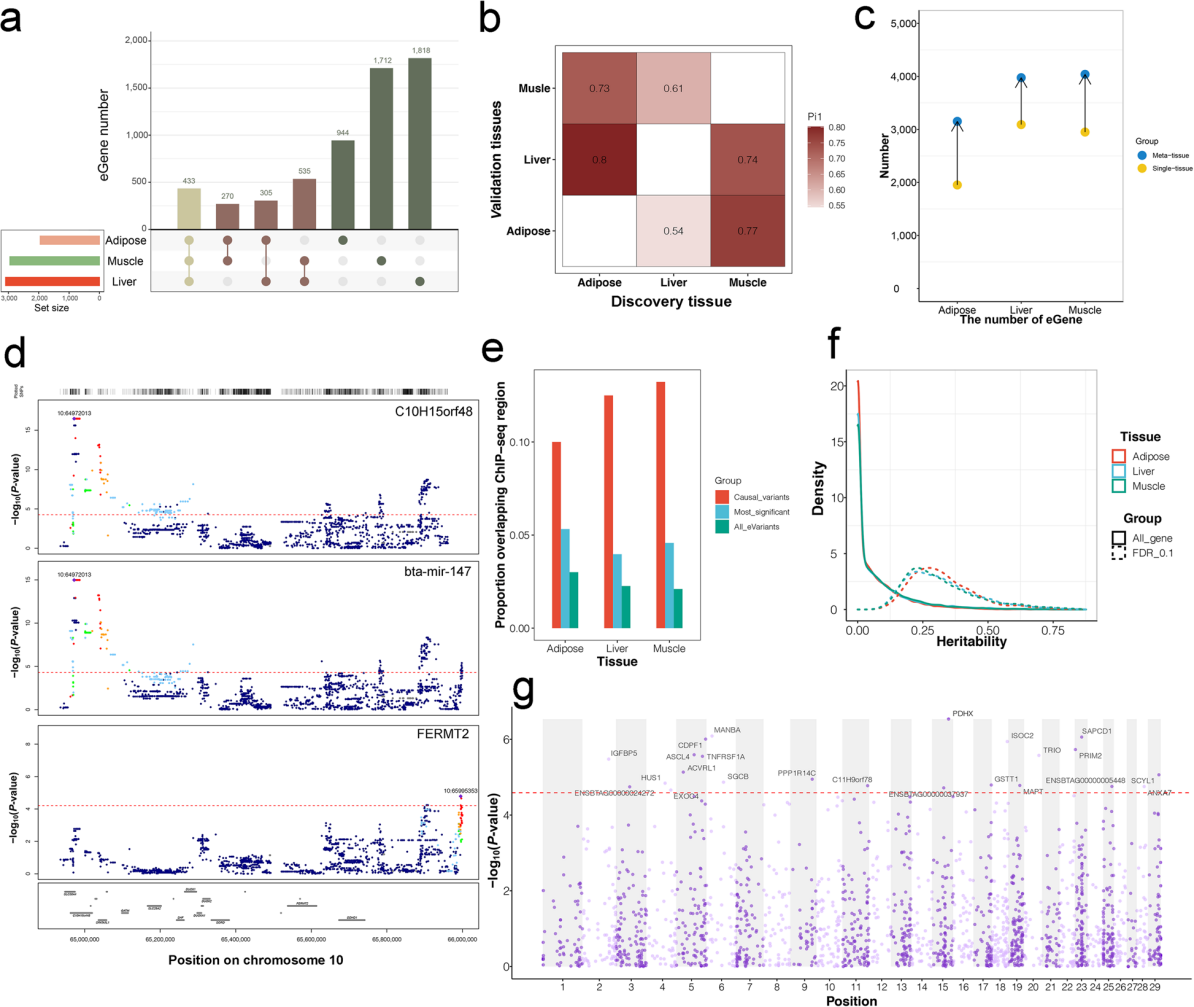
Tissue pattern and pleiotropic of eQTLs, and TWAS. **a** The number of eGenes overlap between tissues. **b** Pairwise sharing patterns of *cis*-eQTL (π_1_ value) across tissues. **c** The increased number of eGenes discovered by multi-tissue *cis*-eQTL analyses.** d** Locuszoom plots of the genetic signals regulating genes at cluster 10:64,961,096–65,908,904. The nominal *P*-values of all local variant-gene associations regarding *C10H15orf48*, *bta-mir-147*, and *FERMT2* were shown. The colors of variants are based on their LD with the most significant eVariant. **e** The proportion of eQTLs (*y*-axis) with chromatin states using fine mapping eVariants, top significant eVariants and total eVariants. **f** Distribution of *cis-H*^*2*^. The solid line corresponds to all tested genes, while the dashed lines are *cis*-heritable genes. **g** Manhattan plot of TWAS between muscle gene expression and daily gain weight

We discovered that any two tissues shared 14.9% of *cis*-eQTLs, 25.6% of eGenes, and 25.7% of eVariants (Fig. 4a and Fig. S13a and b), with strong correlations between effects in all tissues examined (Fig. S14). The effects with aFC values of tissue-shared eQTLs were larger than those of tissue-specific eQTLs (Fig. S13c). To evaluate the *cis*-eQTL sharing patterns between tissues, we calculated π_1_ statistics for each tissue pair. The average π_1_ value was 0.7 (range of 0.54–0.8, Fig. 4b and Fig. S13e). The 433 eGenes shared by all three tissues were involved in immune responses and metabolic pathways (Fig. S13d). The 618,049 tissue-specific *cis*-eQTLs (85% of all *cis*-eQTLs) were examined to determine whether they exhibited consistent directionality in tissues in which they had not yet been identified as eQTLs. With decreasing *P*-value criteria, we observed a progressive decrease in consistency for the second tissue from 100% to near 50% (Fig. S13f and g). A further 108,308 *cis*-eQTLs (15%) that had near-threshold *P*-values in one tissue (*P < *0.001) and were significant in another tissue demonstrated 95% consistency, suggesting that even looser *cis*-eQTL discovery thresholds would still yield additional significant eQTLs. We observed an improvement in *cis*-eQTL discovery using multi-tissue eQTL mapping. The increase rates of eGene discovery were 61.5%, 28.6%, and 36.9% for adipose, liver, and muscle, respectively (Fig. 4c).

On average, 13.9% of *cis*-eQTLs and 9.7% of *trans*-eQTLs were associated with at least two eGenes (Fig. S15b and c). The most prominent *trans*-eQTL with pleiotropy was the T > A substitution at position chr6:95,483,706, which was associated with eight genes in muscle (Fig. S15a). We detected an average of 33.3% *trans*-eQTLs that overlapped with *cis*-eQTLs. We identified 15, 54 and 43 *cis*-eQTL clusters in adipose, liver and muscle, respectively (Fig. S15d). Cluster size varied widely from 47 bp to 3,860,164 bp (Table S4). Interestingly, we found 7 *cis*-eQTL clusters that were shared in all three tissues. We analyzed the colocalization of eQTL signals for each gene pair within a regulatory cluster using Coloc [46]. A posterior probability > 0.8 was applied to identify eGenes regulated by the same genetic signal. We detected 65 adipose gene pairs in 8 clusters, 137 liver gene pairs in 29 clusters and 151 muscle gene pairs in 18 clusters that had colocalized eQTL signals (Table S4). For example, the regulatory cluster 10:64,961,096–65,908,904 in muscle included four eGenes but had multiple genetic signals (Table S4). One of them affects *C10H15orf48* (chromosome 15 open reading frame 48) and *bta-mir-147*, as evidenced by the colocalization probability > 0.95. *FERMT2* (FERM domain containing kindlin 2) had colocalization probabilities < 0.12 with *C10H15orf48* and *bta-mir-147*, leading us to believe that they were regulated by their distinct genetic signals (Fig. 4d). The largest cluster was detected in adipose, which contained 47 genes of major histocompatibility complex (MHC) family with a complex posterior probability (Fig. S15e).

### Fine mapping and TWAS

Fine mapping analysis revealed 111 eQTLs in adipose, 192 eQTLs in liver, and 194 eQTLs in muscle with a posterior probability > 0.8 (Fig. S16a–c and Table S5). The fine-mapped eVariants were more enriched in the histone modification region than the most significant eVariant across all eGenes (Fig. 4e). Gene enrichment analysis showed that these fine-mapped eGenes in liver and adipose were involved in metabolic pathways, whereas the fine-mapped muscle eGenes were enriched in oxidoreductase activity (Fig. S16d–f). The MHC-involved immune pathways were detected in all three tissues. We obtained 2,057, 1,945, and 1,197 *cis*-heritable genes for liver, muscle, and adipose, respectively (Fig. 4f). Using a reference panel of genotype-transcriptome generated from 120 cattle, we predicted the expression of *cis*-heritable genes for the GWAS population and associated gene expression with the 21 traits. We found 805 significant gene-trait pairs for 11 traits in cattle, representing 722 unique genes (Table S6). Among them, the expression of *IGFBP5* (insulin-like growth factor binding protein 5) in muscle was associated with daily weight gain (Fig. 4g). The expression of *LPIN2* (lipin 2) in liver was associated with backfat thickness (Fig. S17).

### The eQTLs help interpret GWAS loci

**Fig. 5:**
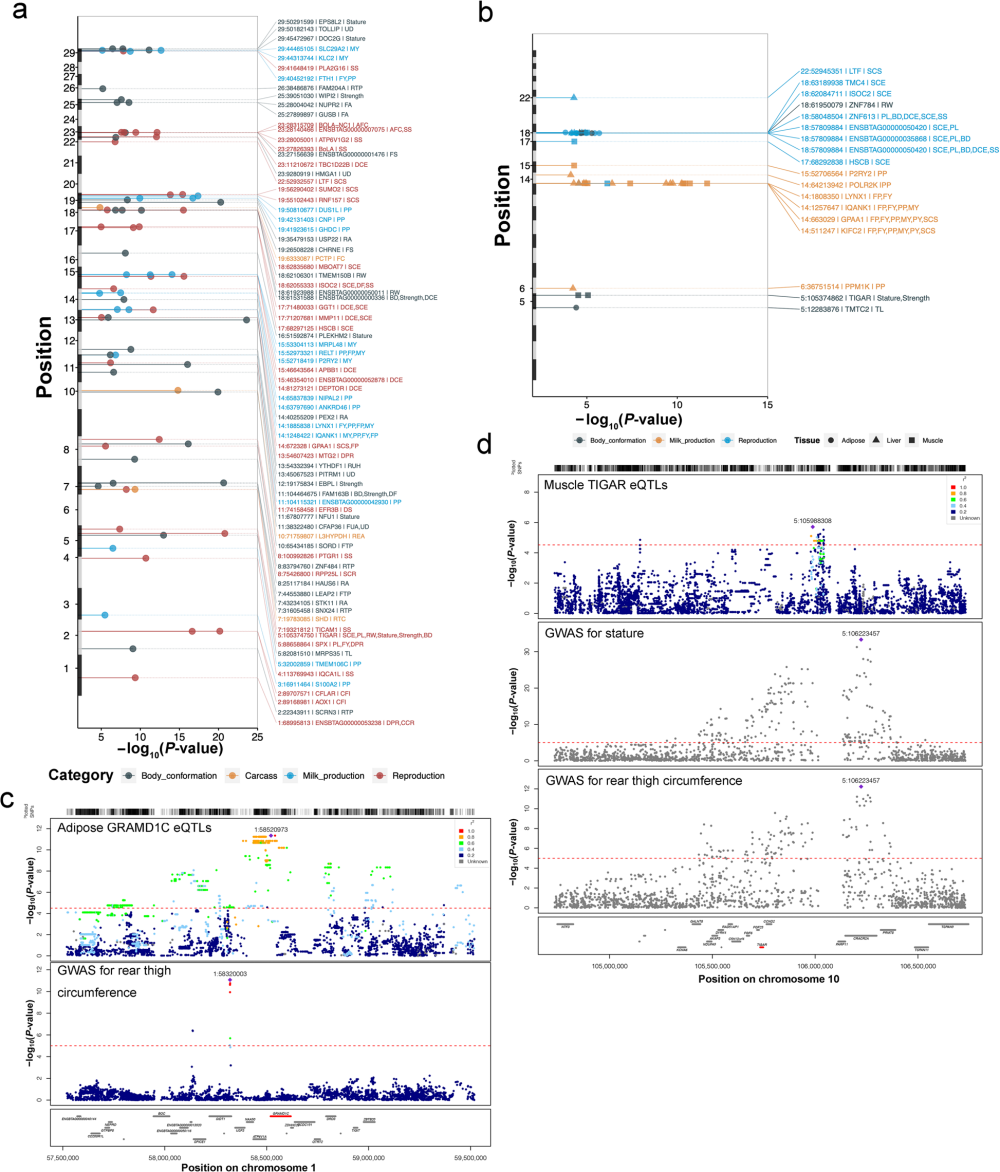
The colocalization of eQTLs and GWAS loci. **a** Manhattan plot showing the colocalization results (H4 > 0.8) between eQTL and GWAS signals. The *x*-axis is the *P*-value of lead eQTLs (points) across traits (colors) in muscle. **b** Manhattan plot showing SMR *P*-value between GWAS signals and eQTLs in different traits (colors) and tissues (point shape). **c** An example of GWAS–eQTL colocalization for *GARMD1C* in adipose. The colors of variants are based on their LD with the most significant variant. **d** An example of GWAS–eQTL colocalization for *TIGAR* in muscle

**Fig. 6:**
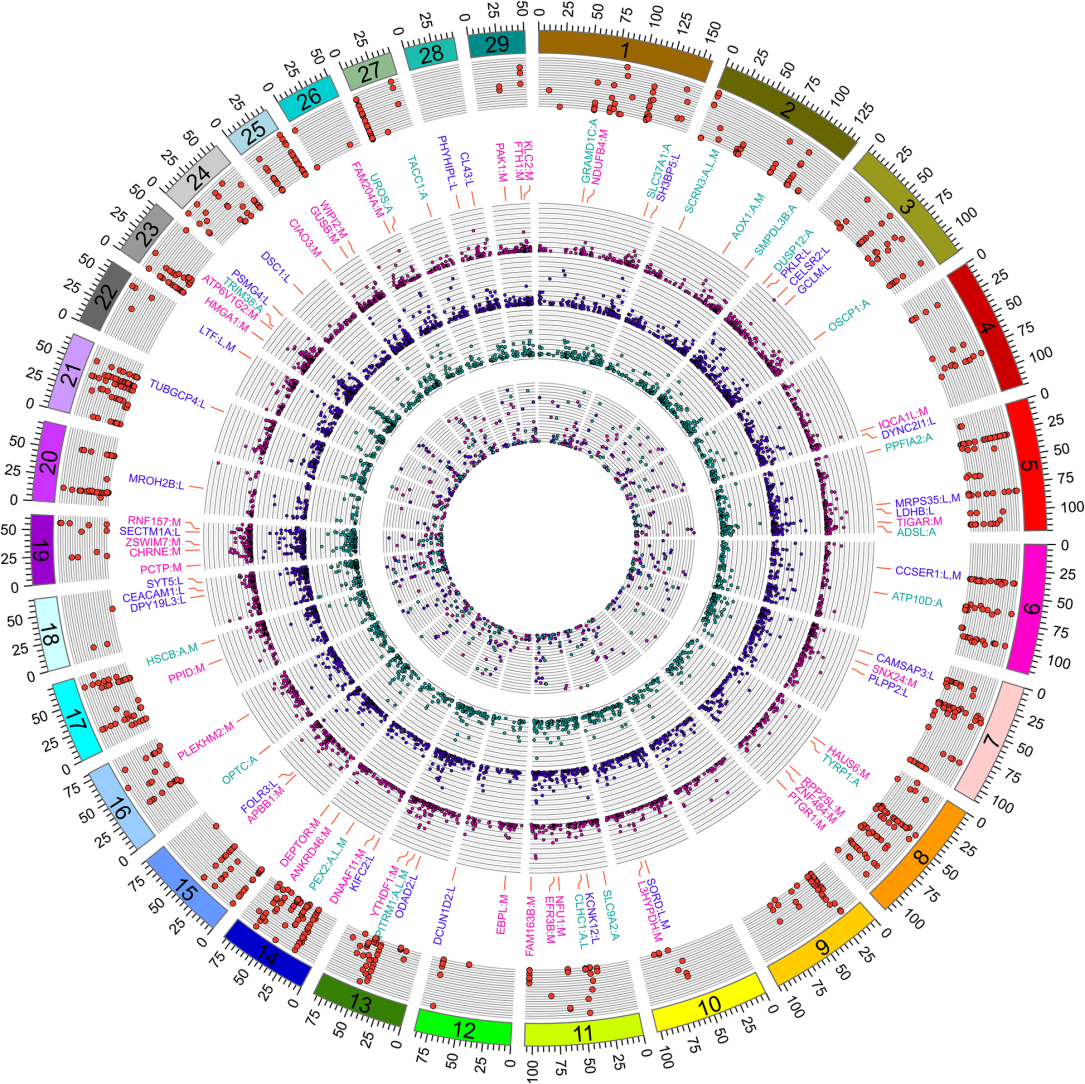
The circos plot of multi-omics significant signatures. The four Manhattan plots with grey backgrounds from outside to inside indicate the significant signatures identified by GWAS, *cis*-eQTL Mapping, and TWAS. The results of GWAS-eQTL colocalization by coloc or SMR are shown between GWAS and eQTL Manhattan plot, which were labeled with gene name and tissue abbreviation after a colon (A: adipose, L: liver, M: muscle). The gene label colors and the dot colors of the eQTL Manhattan plot with green, hot pink, and purple represents adipose, liver, and muscle, respectively

We used the GWAS summary statistics for our 21 traits and 37 publicly available traits [50]. Coloc revealed that eQTLs for 176 eGenes were colocalized with 47 traits (Fig. 5a; Table S7; Fig. S18 and S19), corresponding to 354 gene-trait pairs. The SMR suggested that the abundance of 17 *cis*-regulated genes mediated the association between genetic variants and 15 agronomic traits, resulting in 44 tissue-gene-trait pairs (Fig. 5b). We discovered 29 tissue-gene-trait pairs that were shared by both Coloc and SMR results. In liver tissue, we found that eQTLs of *NADSYN1* (NAD synthetase 1) were colocalized with GWAS signals of stature. The eQTLs of *NDUFS3* (NADH: ubiquinone oxidoreductase core subunit S3) were associated with PH and rib eye area. The eQTLs of *LTF* (lactoferrin) were associated with somatic cell score. The eQTLs of *KIFC2* (kinesin family member C2) were detected colocalized with milk fat (Fig. S20). In adipose tissue, *GRAMD1C* (GRAM domain containing 1C) is a cholesterol transfer gene. We found the eQTLs of *GRAMD1C* were colocalized with the signals of rear thigh circumference (Fig. 5c). The eQTLs of *TMTC2* (transmembrane O-mannosyltransferase targeting cadherins 2) and *ZNF613* (zinc finger protein 613) were associated with body conformation traits. In muscle tissue, the eQTLs of *TIGAR* (TP53-induced glycolysis and apoptosis regulator) were colocalized with rump width, stature, strength, body depth, sire calving ease, and productive life (Fig. 5d). The eQTLs of *NDUFS3* and *L3HYPDH* (*trans*-L-3-hydroxyproline dehydratase) were associated with daily weight gain and rib eye area, respectively. In general, the use of GWAS, eQTL mapping, TWAS, colocalization, and SMR analysis improved our ability to identify potentially causal genes and comprehend the genetic basis of complex traits in cattle (Fig. 6).

## Discussion

This study provides a comprehensive genetic, transcriptome resource for three primary cattle tissues. We show that transcriptional heterogeneity varies across tissues. We create an eQTL catalog for *longissimus dorsi* muscle, backfat and liver, and validate and extend the list of candidate genes and causal variants contributing to cattle agronomic traits.

Recently, some reports have revealed the loci associated with beef production and quality based on GWAS [53–55], and these studies focused on only a few traits. The summary statistics for beef production and quality are still not available, which prevents a more thorough investigation of colocalized signals and an assessment of potential pleiotropic effects. Our study provided a comprehensive GWAS analysis for 21 economic beef traits. We observed that 1,372 associations were novel in our analysis, while 208 associations were previously reported in the cattle QTLdb [1]. Some traits (e.g., striploin, chunk roll, and tenderloin weight) have never been studied before. One fifth of the QTL regions showed genetic pleiotropy effects. Sentinel SNP chr25:40,477,045 near *CARD11*, which is associated with six carcass traits, has been reported to affect residual feed intake [56].

We observed a high density of signals near the respective gene's TSS, which is confirmed by another study [57]. Consistent with other studies, tissue-shared *cis*-eQTLs exhibit high directional consistency between tissues [58]. Even near threshold (*P < *0.001), eQTLs showed > 95% consistency, suggesting that even looser thresholds can identify additional significant eQTLs. Most eQTLs are tissue-specific, implying that the genetic mechanisms of gene expression are different and complex in different tissues. The *cis*-eQTLs are enriched for regulatory elements of promoter and enhancer, probably reflecting that only a tiny percentage of variants in these extended regions have true regulatory effects [59]. In contrast, the enrichment of *trans*-eQTL in functional domains is limited, reflecting the low power of *trans*-eQTL discovery due to the small sample size. Similar to human GTEx V8, the *cis*-eQTLs mediate about one-third of *trans*-eQTLs [59]. The eQTL mapping using multiple tissues has increased power by explicitly modeling tissue-to-tissue sharing patterns [60, 61]. We combined the three tissues in a meta-analysis to improve the power of eQTL mapping, which increased eGene discovery by 43.5% on average.

Compared with the cGTEx results, we detected more than one-third of the eGenes were newly identified, especially for muscle and adipose. One reason for this is the tissue difference: the cGTEx used a variety of muscle and adipose types and the results were not specific for the *longissimus dorsi* muscle and backfat. The second reason is the breed difference: cGTEx used multiple breeds to perform the eQTL mapping, and most cattle were Holsteins (44.5%), while Simmental represented only 1% [21]. The third reason is the SNP data difference. Although cGTEx had efficient performance in detecting eQTLs by RNA-seq alone, some SNPs located in non-transcripted regions would not be detected, which might affect the final detection of eQTLs. Therefore, eQTL mapping needs to be performed in a more refined tissue and a specific breed in the future.

Our results provide important insights into the pleiotropy of variants. The eVariants regulate multiple genes, and this phenomenon occurs clustered in distinct regions of the cattle genome. The largest of these clusters contains at least 47 eGenes of the MHC region, which is essential for immunological response [62]. This cluster was also detected in liver and muscle tissues, even in human retinal tissue [63], implying that eQTLs are widely distributed in MHC regions. The fine-mapping results of *cis*-eQTL offer a collection of hundreds of functional variants that are most likely causative for eGenes. This TWAS strategy reveals hundreds of genes for which changes in genetically predicted expression are associated with 11 agronomic traits. *IGFBP5* is a crucial focal regulator of the local action of IGF-I [64] by sequestering the growth factor and influencing protein accretion and myoblasts development [65, 66]. *IGFBP5* was detected to be related to daily weight gain. *LPIN2* played a significant part in regulating fatty acid metabolism at various levels that were associated with type 2 diabetes and fat distribution [67, 68]. We found *LPIN2* was related to backfat thickness*.* The tubulin alpha-1D chain (*TUBA1D*) is involved in cytoskeletal organization and cell motility [69]. Unc-45 myosin chaperone B (*UNC45B*) is involved in myocyte maturation and striation formation [70]. The expression of *TUBA1D* and *UNC45B* in muscle was related to rib-eye weight.

Colocalization of GWAS signals and *cis*-eQTLs in three tissues helps identify causal genes for hitherto unsolved association signals. Multiple GWAS signals were detected to colocalize with *cis*-eQTLs in three tissues. The eQTLs of *NADSYN1*, *NDUFS3*, *LTF* and *KIFC2* in liver were related to height, PH and Rib eye area, somatic cell score and milk fat, respectively. *NADSYN1* is related to calcium metabolism [71, 72]. *NDUFS3* was correlated with the juiciness and flavor of beef in a previous study [73]. *LTF* is a milk glycoprotein favorably associated with the immune system of cows [74]. *KIFC2* is involved in vesicle-mediated transport [75]. The eQTLs of *GRAMD1C* in adipose were related to rear thigh circumference. *GRAMD1C* is a cholesterol transfer gene that contributes to cholesterol transfer activity [76]. Both *TMTC2* and *ZNF613* were GWAS fine-mapped candidate genes for body conformation traits in a previous study [50]. In this study, it was confirmed that the variants can alter the expression of *TMTC2* and *ZNF613*, which affects body conformation traits.

*TIGAR* can improve mitochondrial functions and reduce muscle cell autophagy [77, 78], which was associated with body weight and stature in cattle [79, 80]. We detected the eQTLs of *TIGAR* were related to stature, strength, and reproductive traits. *NDUFS3* contributes to energy metabolism of transformed cells [81]. Deletion of *NDUFS3* gene in muscle would induce myopathy phenotype in mice [82]. *L3HYPDH* was a candidate gene associated with the rib eye area [83], which was confirmed by our eQTLs results. These results prove essential for acquiring a greater understanding of the molecular mechanisms of a specific trait by considering gene expression, which includes the functional characteristics of genes related to various traits. The GWAS signals associated with eQTL serve as a starting point for further research in beef cattle. In addition, this availability of eQTL data from *longissimus dorsi* muscle, backfat and liver, provides a resource for resolving additional genetic association signals emerging from ongoing extensive efforts to improve production and quality traits of cattle, for which these three tissue types are significant.

## Conclusions

Interpretation of the genetic mechanism of complex traits based on molecular phenotype of primary tissues is relatively late in beef cattle. We demonstrate an efficient multi-omics data strategy for agronomic traits using three primary tissues of beef cattle, which can serve as a valuable approach that moves from fundamental discovery to decipher genetic mechanisms of complex traits of cattle. By integrating eQTL and GWAS data, we constructed a molecular QTL map in cattle that helps to resolve genetic association signals by detecting candidate genes such as *KIFC2*, *TIGAR* and *GRAMD1C*. Moreover, these new candidate genes or causal DNA variants will help improve genomic prediction accuracy and the benefit of genetic improvement programs in beef cattle [84, 85].

## Supplementary Information

**Additional file 1: Fig. S1.** The sample size of primary tissue in beef cattle. The number of individuals with data for each tissue. The 770K SNP arrays of 120 individuals were imputed to whole-genome SNPs using the 1,847 multiple-breed cattle reference panel.

**Additional file 2: Fig. S2.** RNA sequencing information. **a** The proportion of the mapped reads of RNA sequencing in the genome region. Most Reads were mapped to the coding areas. **b** The clean reads and mapping rate of 356 samples. The average clean-read number and mapping rate were 45.6 million and 97%, respectively.

**Additional file 3: Fig. S3.** Hierarchical clustering of 356 samples. The clustering of 356 samples shows that the tissue is the most differentiated among all samples. The batch factor could affect several samples. The sample difference caused by age was limited.

**Additional file 4: Fig. S4.** Characterization of PEER factors and PCA of genotypes. **a**–**c** Factor weight variances are computed up to 30 factors in three tissues. Factor weight variances were stable for all three tissues when the number of PEER factors reaches five. **d** PCA of genotype data of 120 cattle.

**Additional file 5: Fig. S5.** Biological covariates analysis. **a**–**c** Percentage of variance explained by covariates in each of the three tissues. Data are presented as a percentage (%) of the total variance explained. **d**–**f** Pairwise correlation between the covariates. Red and white indicate high and low correlation, respectively.

**Additional file 6: Fig. S6.** Correlation of gene co-expression modules with agronomic traits in liver and adipose. The modules were denoted by different colors. Correlation of module eigengene with each agronomic trait displayed in the corresponding box (top: coefficient, bottom: *P*-value). The color of each box represents a positive correlation (red) or a negative correlation (blue).

**Additional file 7: Fig. S7.** The summary of *cis*-eQTL and *trans*-eQTL results.

**Additional file 8: Fig. S8.** Manhattan plot of *cis*-eQTLs in liver and backfat. **a** Manhattan plot showing the nominal *P*-value (*y*-axis) for all *cis*-eQTLs in the liver. **b** Manhattan plot of backfat *cis*-eQTLs.

**Additional file 9: Fig. S9.** Dot plot showing the locations, *P*-value and effect sizes for all significant *trans*-eQTL in liver and adipose. **a** Locations of *trans*-eQTL in liver. Variants and gene positions are shown on the *x*-axis and *y*-axis, respectively. Each dot was a significant *trans*-eQTLs (FDR < 0.05). The size of each dot represents the -log10 scaled *P*-values. The color of each dot represents the direction of slope effect. **b** Locations of *trans*-eQTL in adipose (backfat).

**Additional file 10: Fig. S10.** *P*-value distributions of most significant *cis*-eQTLs per gene relative to TSS in three tissues. The scatter plots show the -log_10_ (*P*-values) of the most significant SNP per gene for eGenes and their distance to the TSS (in kilobases).

**Additional file 11: Fig. S11.** The independent eQTLs. **a** The number of independent signals in three tissues. **b** The primary signals (golden) and the secondary signals (blue) relative to TSS are shown using point plot (left). The right plot is a boxplot of the absolute distance of primary and secondary signals to the TSS. The Wilcoxon test is used to compute significance. **c** The distance to TSS for adipose tissue.

**Additional file 12: Fig. S12.** The characters of eQTLs. **a** The SNP-heritability compares between eGene and non-eGene. **b**–**d** The most significant *P*-value of *cis*-eQTLs per gene with function of their heritability for adipose, liver and muscle. **e** The genomic location enrichment of the top significant *cis*-eQTLs (red dot) and all significant *cis*-eQTLs (blue dot) in three tissues. **f** The gene expression comparison between eGenes and non-eGenes.

**Additional file 13: Fig. S13.** *Cis*-eQTLs across tissues. **a** Venn diagram of *cis*-eQTLs pairs across three tissues. **b** Venn diagram of eVariants across three tissues. **c** The effect compares specific and shared *cis*-eQTLs. **d** The pathway analysis of common tissue is shared by three tissues. **e** The *P*-value distribution of the shared eQTL of tissue 1 in tissue 2. **f** The eQTL effect size of the tissue-2 (*y*-axis) increases with the significance of *P*-value (*x*-axis) for both positive and negative effect tissue-1 eQTLs. **g** The directionality consistency of the shared eQTL increases with the significance of the *P*-value (*x*-axis) for both positive and negative effect tissue-1 eQTLs.

**Additional file 14: Fig. S14.** The correlation of eQTL slope effect sizes between tissues. **a** Correlation between muscle and adipose. **b** Correlation between muscle and liver. **c** Correlation between liver and adipose. **d** Correlation of common eQTL effect sizes across all three tissues using three dimensions. **e** Correlation matrix plot of common eQTL effect sizes across three tissues.

**Additional file 15: Fig. S15.** The pleiotropic character of eQTLs. **a** The circos plot of the *trans*-eQTL pleiotropic example. **b** The number of *cis*-eGenes regulated by eVariant. **c** The number of *trans*-eGenes regulated by eVariant. **d** The distribution of eQTL clusters in the cattle genome. The green, yellow and red highlights represent muscle, adipose and liver, respectively. **e** Data showing the colocalization posterior probabilities (H4) for all 47 eGenes located in cluster 23:27612341-31472505 in adipose. H4 was colored in red with increasing intensity.

**Additional file 16: Fig. S16.** The fine-mapping results of *cis*-eQTLs by DAP-g. **a**–**c** The posterior probability distribution of *cis*-eQTL fine mapping using DAP-g in adipose, liver, and muscle. **d**–**f** Gene enrichment for these genes with fine-mapping variants in adipose, liver, and muscle.

**Additional file 17: Fig. S17.** Manhattan plot of TWAS between liver gene expression and backfat thickness. Manhattan plot showing the genomic position (*x*-axis) and the *P*-value for association (*y*-axis).

**Additional file 18: Fig. S18.** Manhattan plot showing the colocalization results (H4 > 0.8) between eQTL and GWAS signals. The x-axis is the *P*-value of lead eQTLs (points) across traits (colors) in adipose.

**Additional file 19: Fig. S19.** Manhattan plot showing the colocalization results (H4 > 0.8) between eQTL and GWAS signals. The x-axis is the *P*-value of lead eQTLs (points) across traits (colors) in liver.

**Additional file 20: Fig. S20.** An example of GWAS–eQTL colocalization for *KIFC2* in the liver. The colors of variants are based on their LD with the most significant variant.

**Additional file 21: Table S1.** The information of GWAS results for 21 agronomic traits.

**Additional file 22: Table S2.** The information of 356 RNA-seq samples and their mapping results.

**Additional file 23: Table S3.** The significant modules-trait pairs in three tissues, and KEEG annotation of significant module genes.

**Additional file 24: Table S4.** Overview of *cis*-eQTL clusters and posterior probability for colocalization of genetic signals underlying eGenes of the same cluster in three tissues.

**Additional file 25: Table S5.** Overview of fine-mapping results in three tissues.

**Additional file 26: Table S6.** TWAS results for 11 agronomic traits in three tissues.

**Additional file 27: Table S7.** The results of GWAS-eQTL colocalization by Coloc or SMR in three tissues.

## Data Availability

Full summary statistics of the GWAS for 21 beef cattle traits are available in figshare with the identifier https://doi.org/10.6084/m9.figshare.20032988.v1. All eQTL results are available on GitHub with the identifier https://github.com/WentaoCai/Cattle_eQTL_results. Other results were provided in the Additional file. The RNA-seq that supports this study's findings is available from the NCBI SRA database with accession numbers PRJNA846691 and PRJNA721166. The scripts used for data processing and analyses are available on the website through the following link, https://wentaocai.github.io/eQTL-analysis/.

## Abbreviations

BH: Benjamini-Hochberg
DAP: Deterministic approximation of posteriors
eQTLs: Expression quantitative trait loci
FDR: False discovery rate
GTEx: The Genotype-Tissue Expression
GWAS: Genome-wide associated studies
LD: Linkage disequilibrium
MR: Mendelian randomization
PCs: Principal components
PEER: Probabilistic estimation of expression residuals
REML: Restricted maximum likelihood
SMR: Summary data-based Mendelian randomization
SNPs: Single nucleotide polymorphisms
t-SNE: t-Distributed Stochastic Neighbor Embedding
TSS: Transcription start site
TWAS: Transcriptome-wide association studies
WGCNA: Weighted correlation network analysis
