# Supplementary figures and images for: The eQTL colocalization and transcriptome-wide association study identify potentially causal genes responsible for economic traits in Simmental beef cattle
Source: J Anim Sci Biotechnol. 2023 May 11;14:78. doi: 10.1186/s40104-023-00876-7 (PMC10173583; doi:10.1186/s40104-023-00876-7)

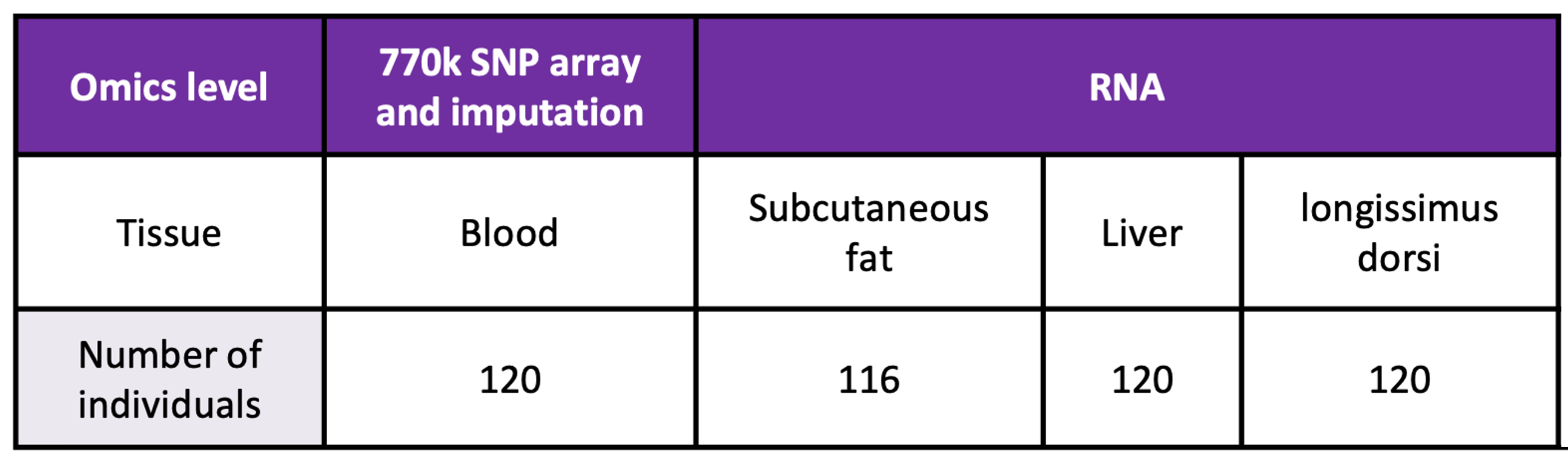

Supplement: Supplementary file 1 — Additional file 1: Fig. S1. The sample size of primary tissue in beef cattle. The number of individuals with data for each tissue. The 770K SNP arrays of 120 individuals were imputed to whole-genome SNPs using the 1,847 multiple-breed cattle reference panel. [file 40104_2023_876_MOESM1_ESM.tif]

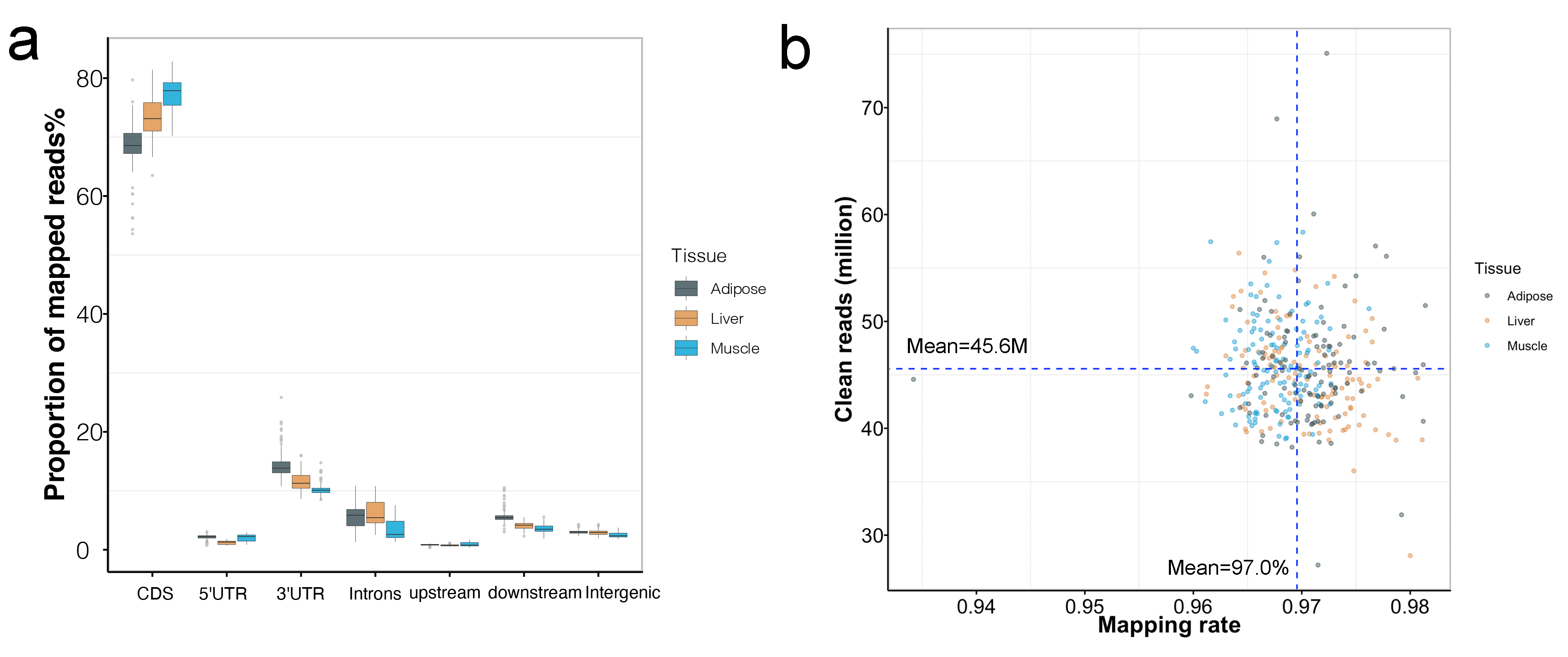

Supplement: Supplementary file 2 — Additional file 2: Fig. S2. RNA sequencing information. a The proportion of the mapped reads of RNA sequencing in the genome region. Most Reads were mapped to the coding areas. b The clean reads and mapping rate of 356 samples. The average clean-read number and mapping rate were 45.6 million and 97%, respectively. [file 40104_2023_876_MOESM2_ESM.tif]

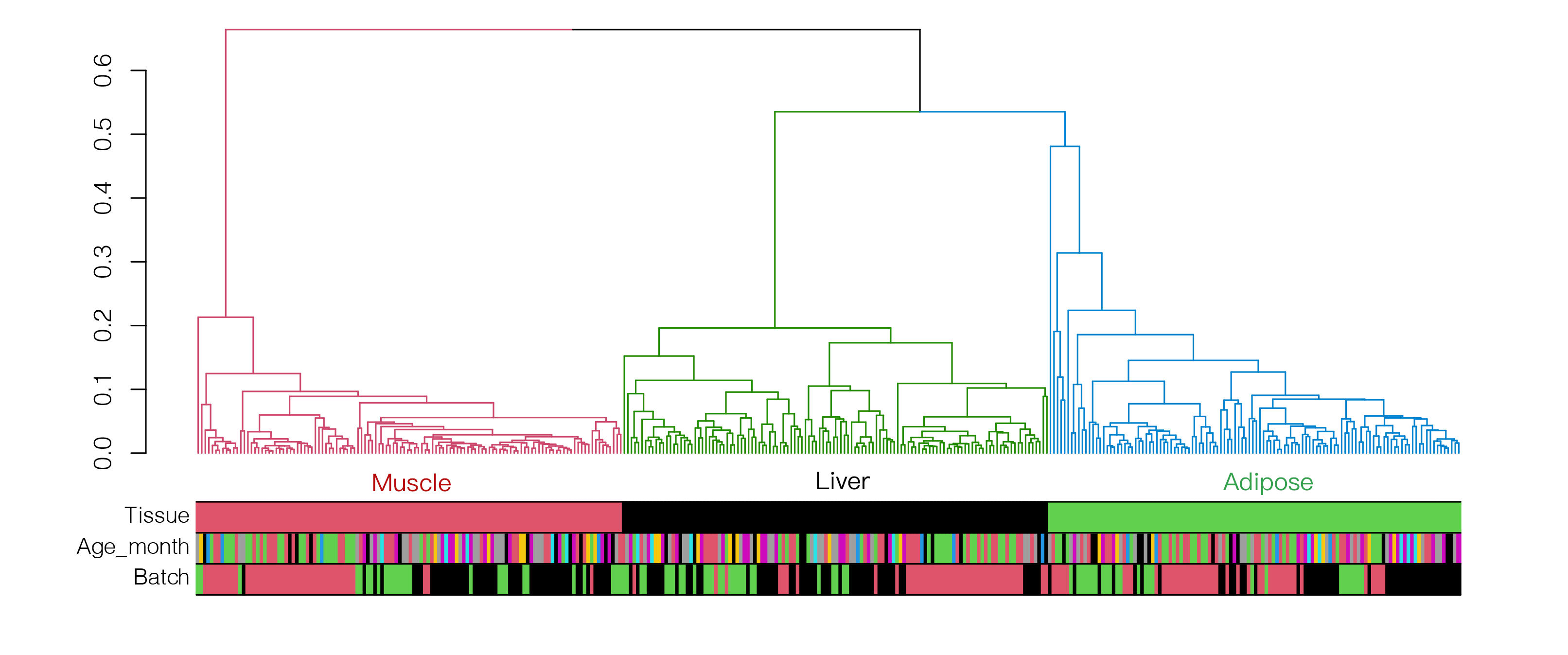

Supplement: Supplementary file 3 — Additional file 3: Fig. S3. Hierarchical clustering of 356 samples. The clustering of 356 samples shows that the tissue is the most differentiated among all samples. The batch factor could affect several samples. The sample difference caused by age was limited. [file 40104_2023_876_MOESM3_ESM.tif]

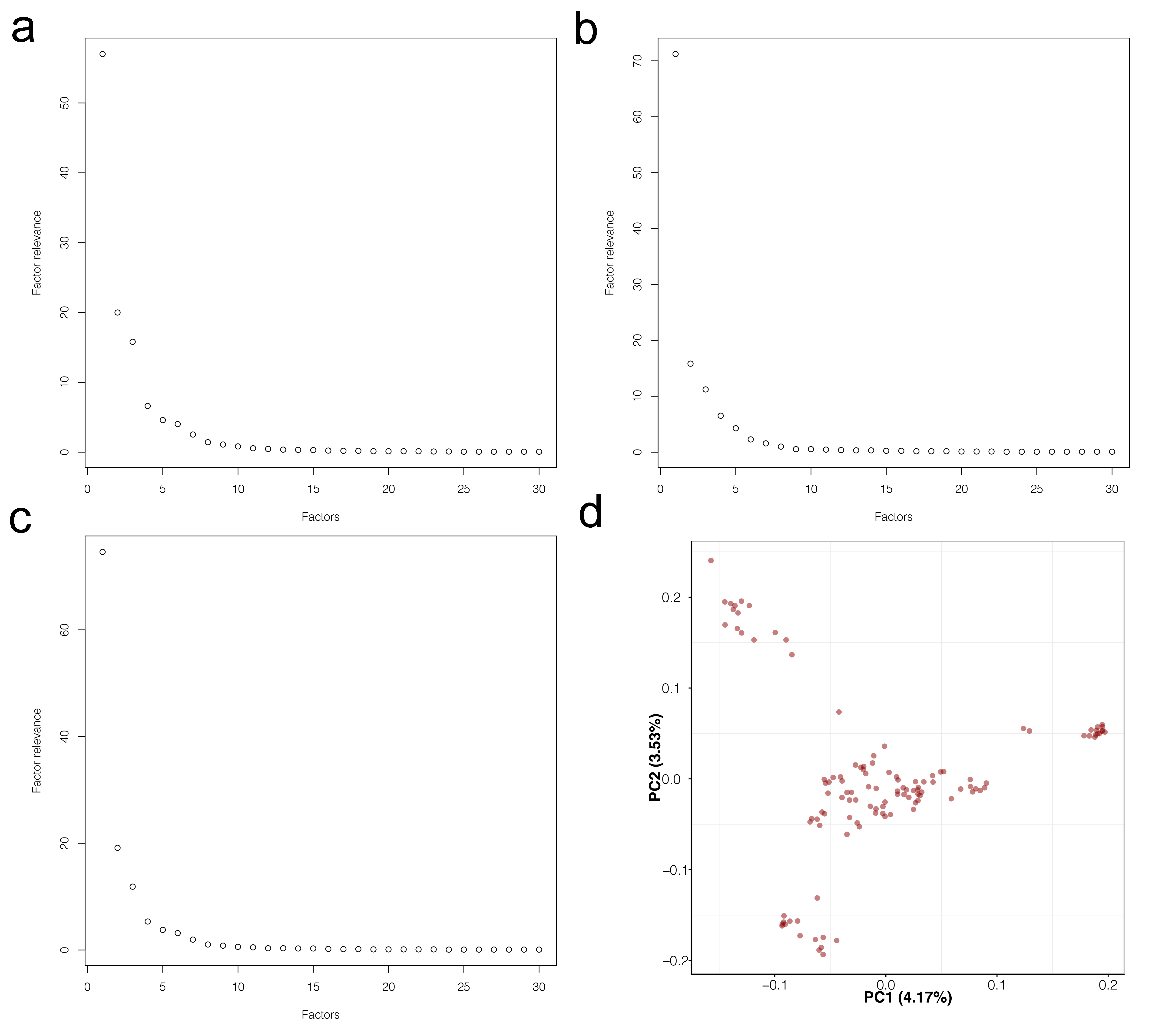

Supplement: Supplementary file 4 — Additional file 4: Fig. S4. Characterization of PEER factors and PCA of genotypes. a–c Factor weight variances are computed up to 30 factors in three tissues. Factor weight variances were stable for all three tissues when the number of PEER factors reaches five. d PCA of genotype data of 120 cattle. [file 40104_2023_876_MOESM4_ESM.tif]

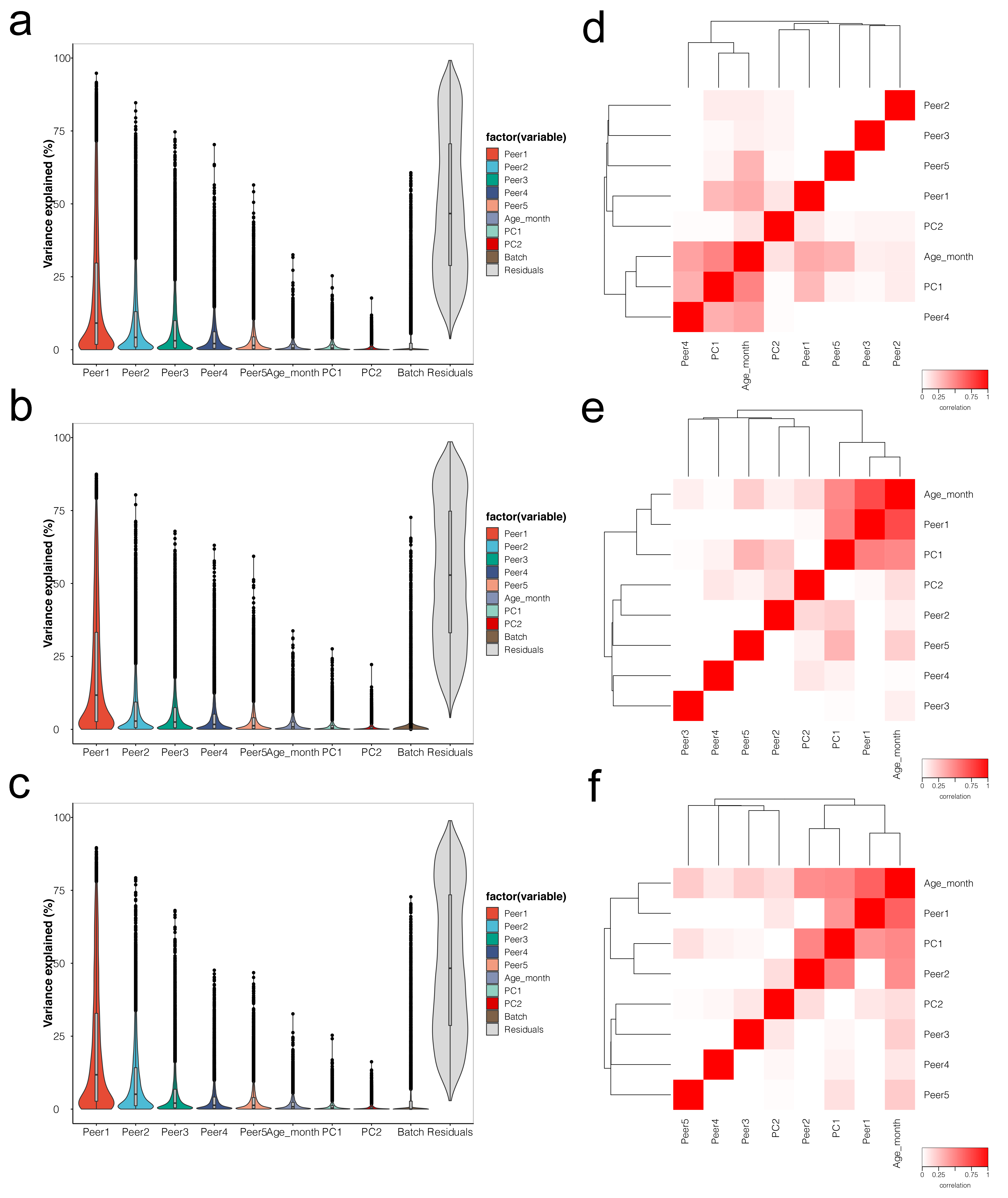

Supplement: Supplementary file 5 — Additional file 5: Fig. S5. Biological covariates analysis. a–c Percentage of variance explained by covariates in each of the three tissues. Data are presented as a percentage (%) of the total variance explained. d–f Pairwise correlation between the covariates. Red and white indicate high and low correlation, respectively. [file 40104_2023_876_MOESM5_ESM.tif]

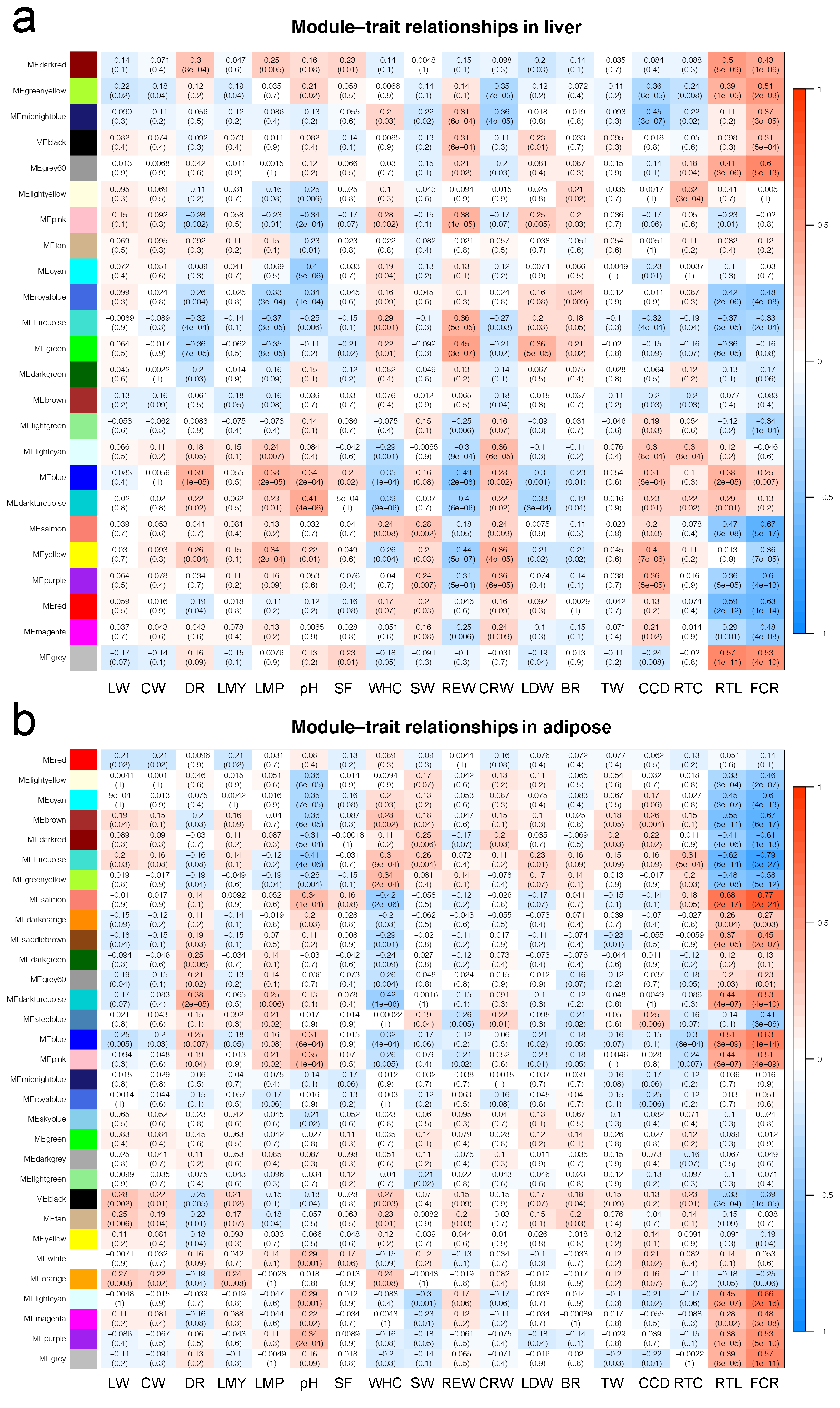

Supplement: Supplementary file 6 — Additional file 6: Fig. S6. Correlation of gene co-expression modules with agronomic traits in liver and adipose. The modules were denoted by different colors. Correlation of module eigengene with each agronomic trait displayed in the corresponding box (top: coefficient, bottom: P-value). The color of each box represents a positive correlation (red) or a negative correlation (blue). [file 40104_2023_876_MOESM6_ESM.tif]

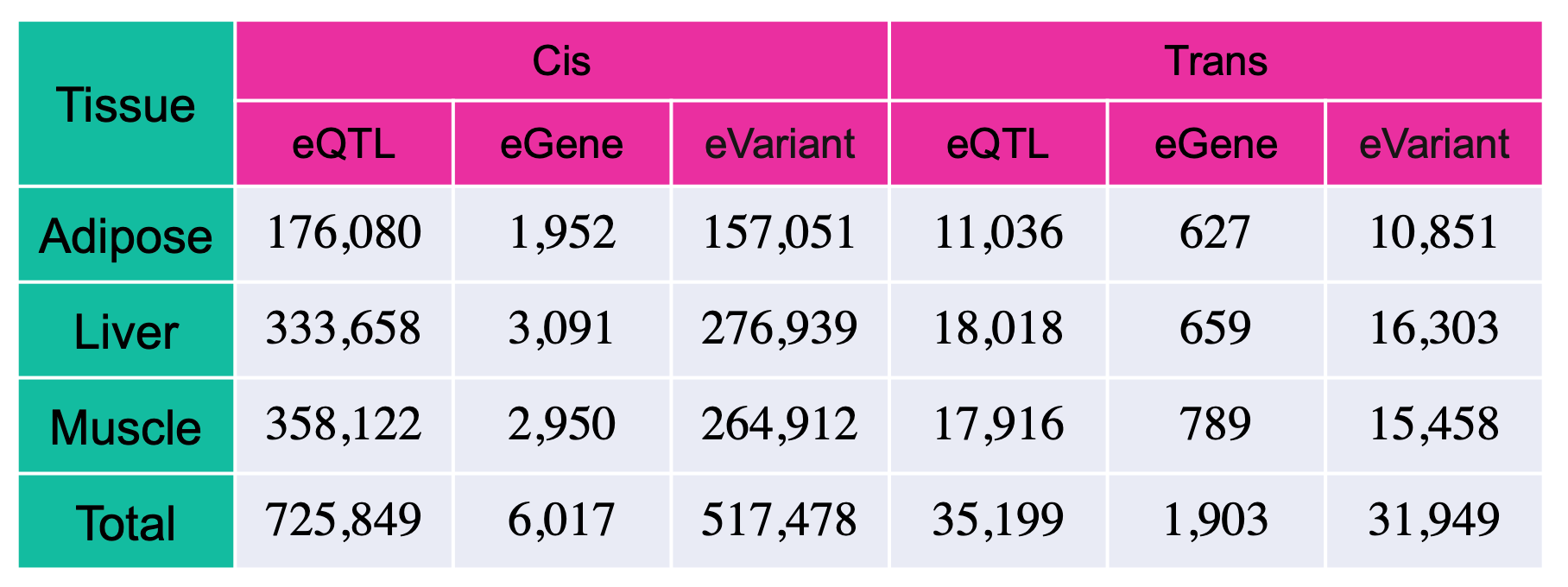

Supplement: Supplementary file 7 — Additional file 7: Fig. S7. The summary of cis-eQTL and trans-eQTL results. [file 40104_2023_876_MOESM7_ESM.tif]

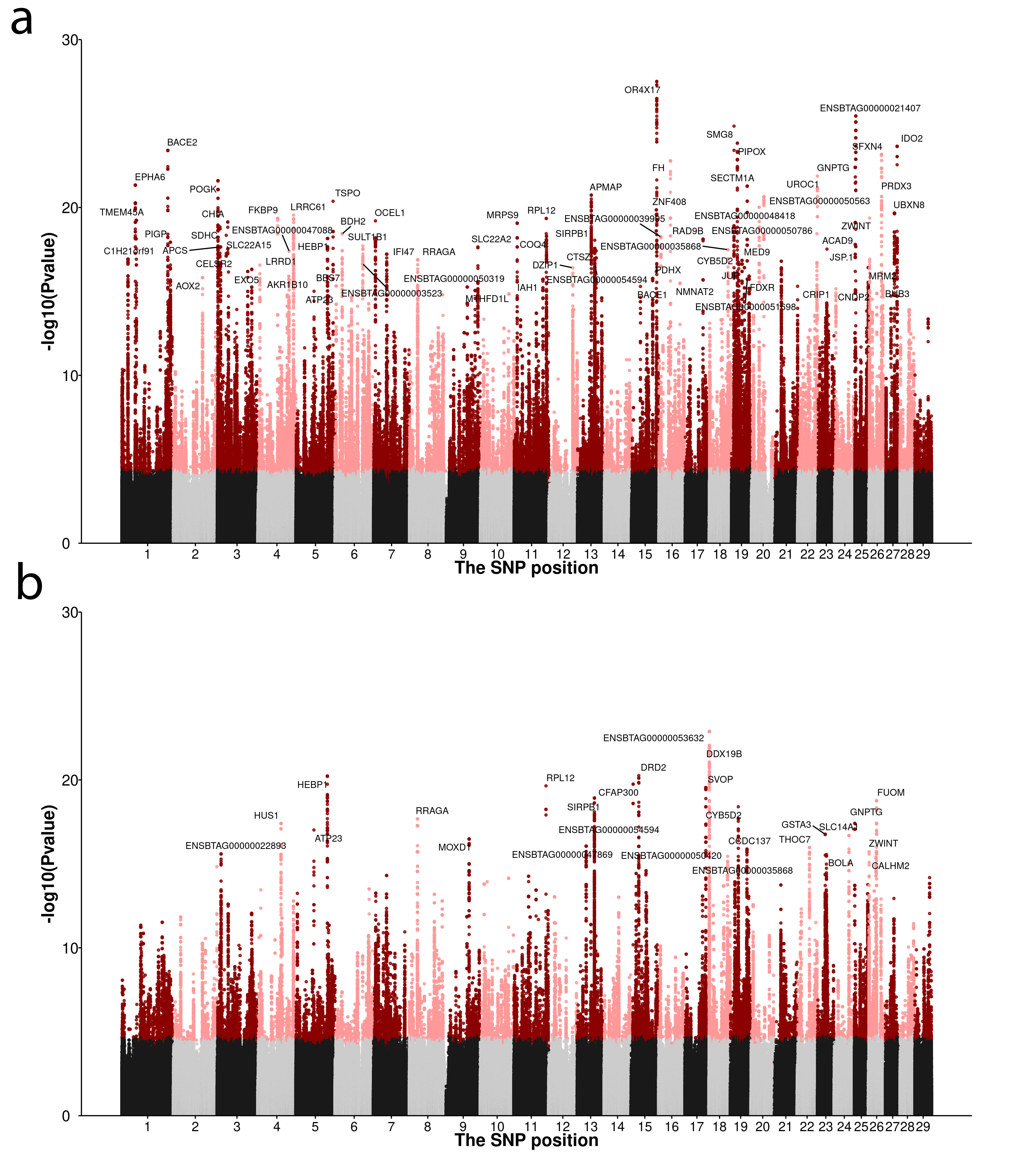

Supplement: Supplementary file 8 — Additional file 8: Fig. S8. Manhattan plot of cis-eQTLs in liver and backfat. a Manhattan plot showing the nominal P-value (y-axis) for all cis-eQTLs in the liver. b Manhattan plot of backfat cis-eQTLs. [file 40104_2023_876_MOESM8_ESM.tif]

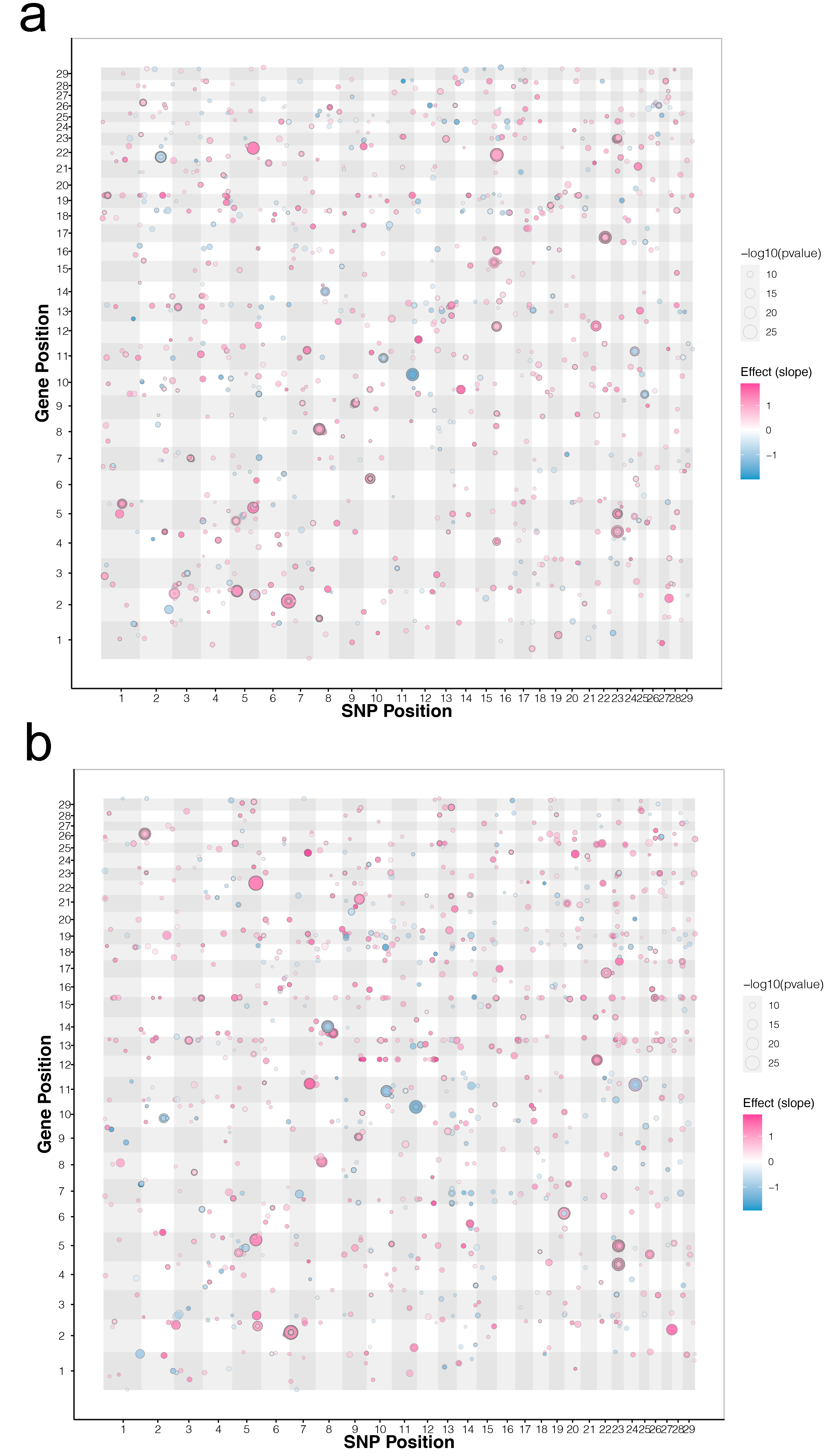

Supplement: Supplementary file 9 — Additional file 9: Fig. S9. Dot plot showing the locations, P-value and effect sizes for all significant trans-eQTL in liver and adipose. a Locations of trans-eQTL in liver. Variants and gene positions are shown on the x-axis and y-axis, respectively. Each dot was a significant trans-eQTLs (FDR < 0.05). The size of each dot represents the -log10 scaled P-values. The color of each dot represents the direction of slope effect. b Locations of trans-eQTL in adipose (backfat). [file 40104_2023_876_MOESM9_ESM.tif]

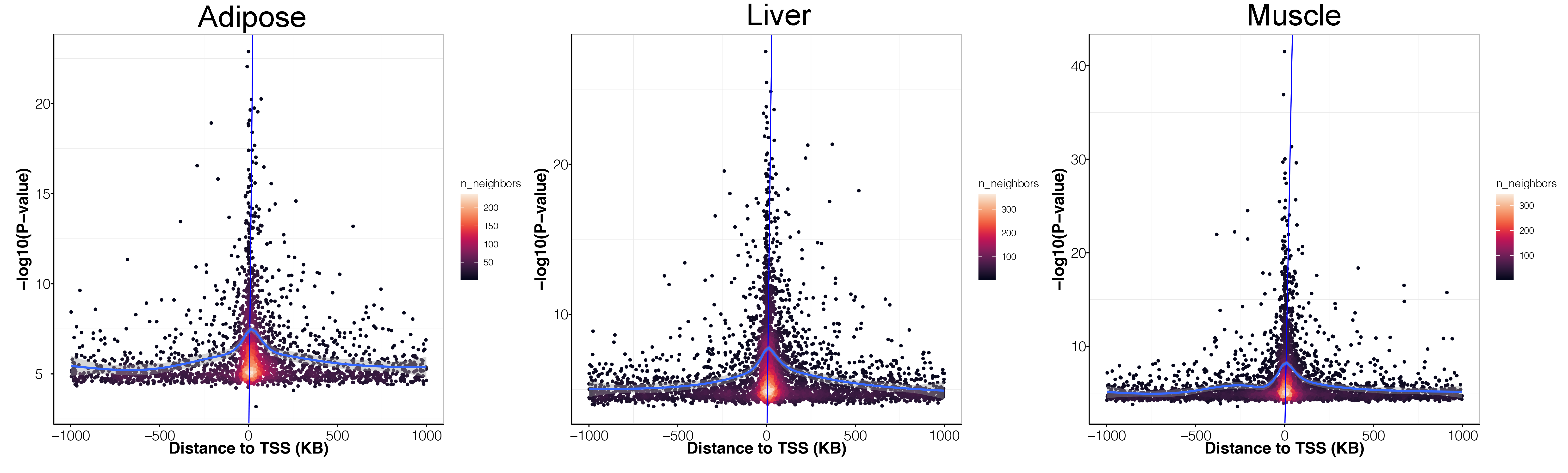

Supplement: Supplementary file 10 — Additional file 10: Fig. S10. P-value distributions of most significant cis-eQTLs per gene relative to TSS in three tissues. The scatter plots show the -log10 (P-values) of the most significant SNP per gene for eGenes and their distance to the TSS (in kilobases). [file 40104_2023_876_MOESM10_ESM.tif]

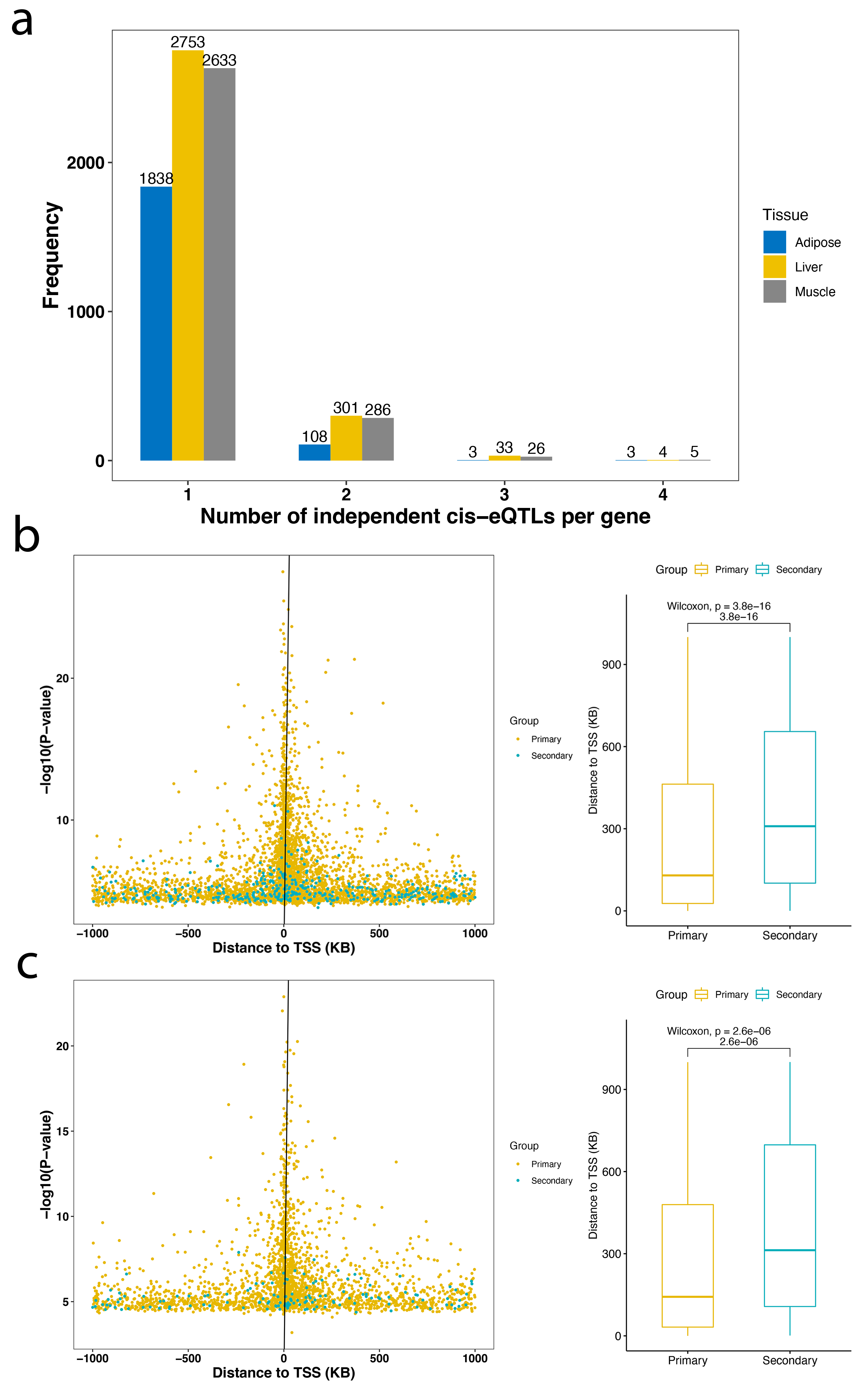

Supplement: Supplementary file 11 — Additional file 11: Fig. S11. The independent eQTLs. a The number of independent signals in three tissues. b The primary signals (golden) and the secondary signals (blue) relative to TSS are shown using point plot (left). The right plot is a boxplot of the absolute distance of primary and secondary signals to the TSS. The Wilcoxon test is used to compute significance. c The distance to TSS for adipose tissue. [file 40104_2023_876_MOESM11_ESM.tif]

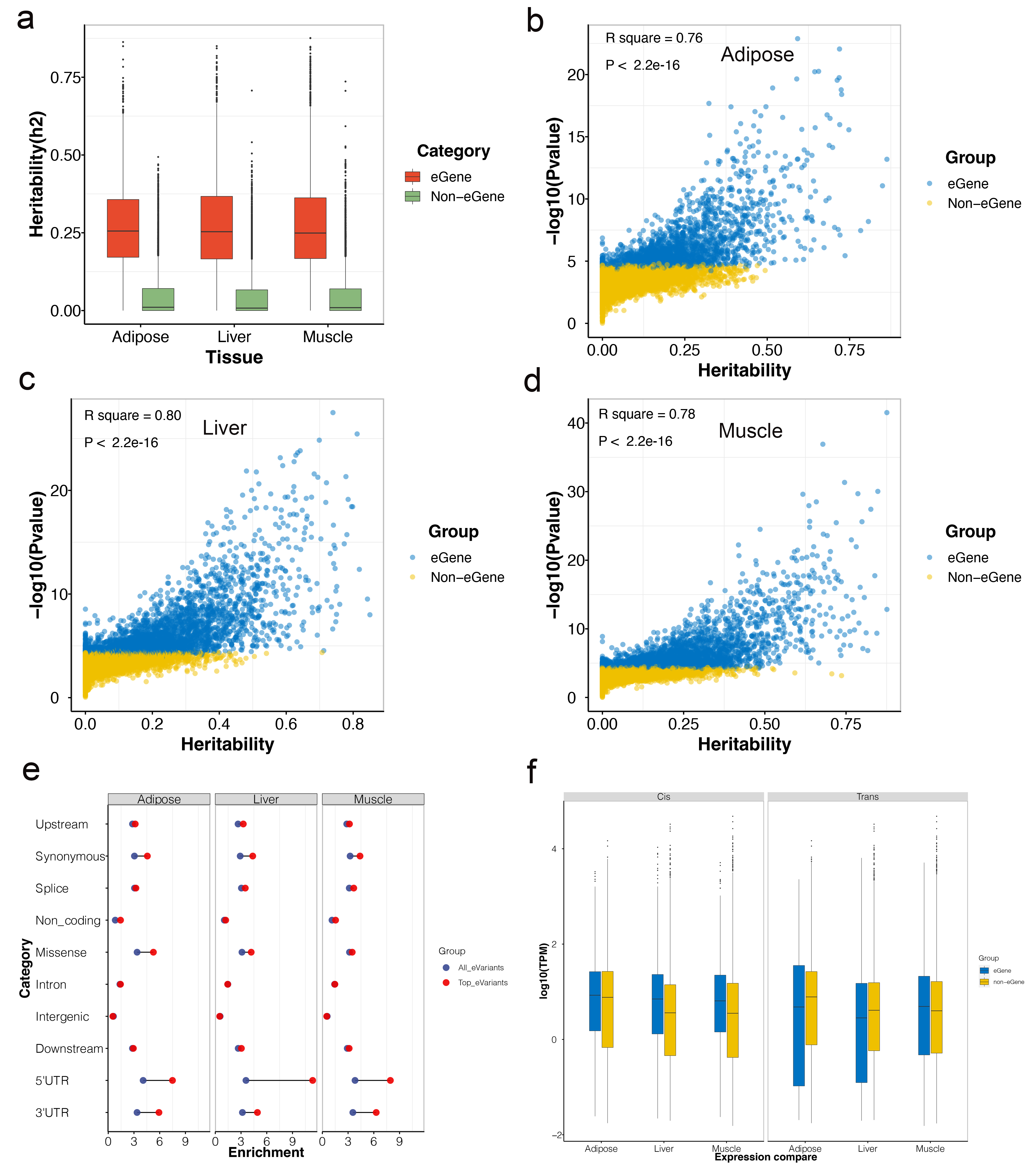

Supplement: Supplementary file 12 — Additional file 12: Fig. S12. The characters of eQTLs. a The SNP-heritability compares between eGene and non-eGene. b–d The most significant P-value of cis-eQTLs per gene with function of their heritability for adipose, liver and muscle. e The genomic location enrichment of the top significant cis-eQTLs (red dot) and all significant cis-eQTLs (blue dot) in three tissues. f The gene expression comparison between eGenes and non-eGenes. [file 40104_2023_876_MOESM12_ESM.tif]

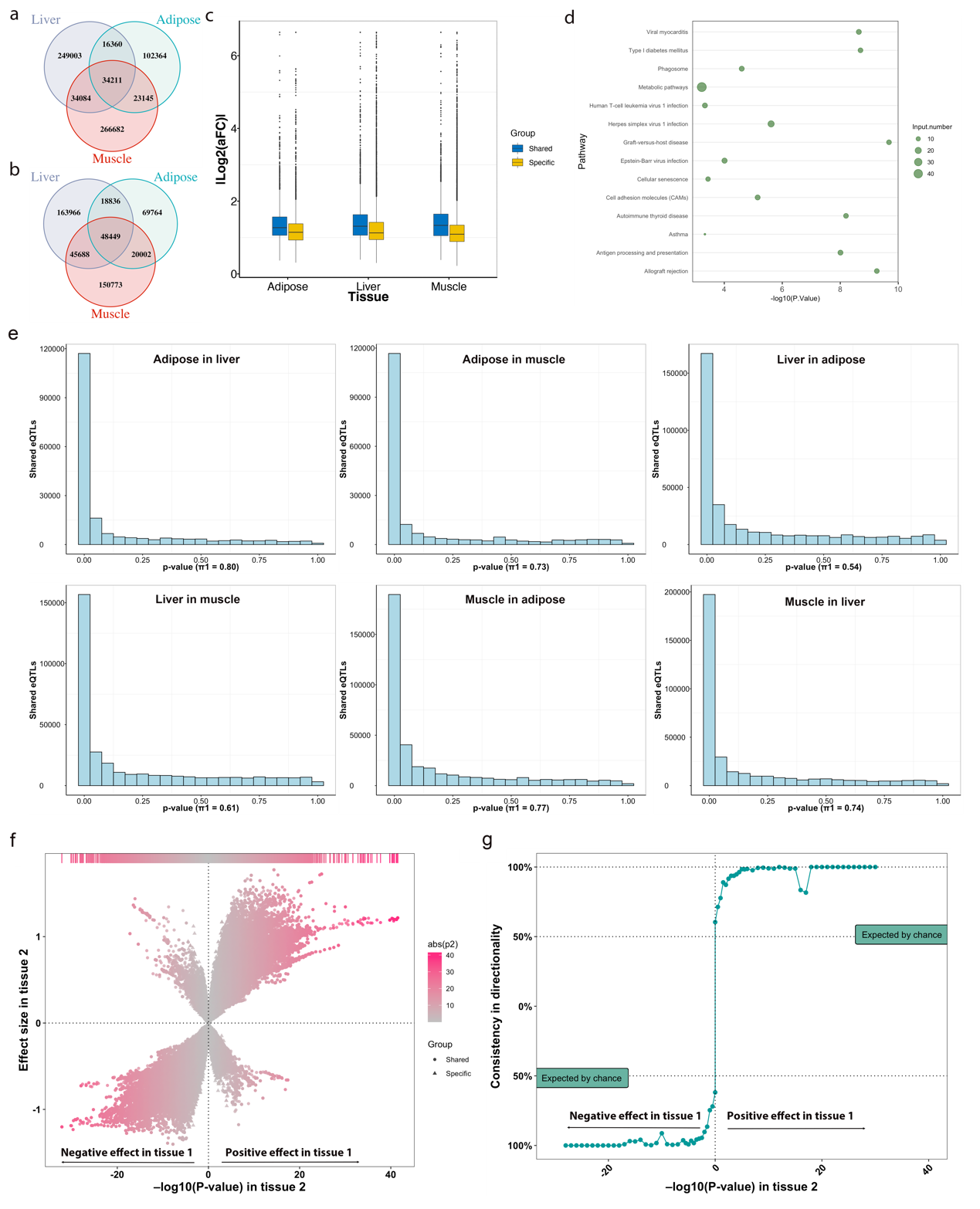

Supplement: Supplementary file 13 — Additional file 13: Fig. S13. Cis-eQTLs across tissues. a Venn diagram of cis-eQTLs pairs across three tissues. b Venn diagram of eVariants across three tissues. c The effect compares specific and shared cis-eQTLs. d The pathway analysis of common tissue is shared by three tissues. e The P-value distribution of the shared eQTL of tissue 1 in tissue 2. f The eQTL effect size of the tissue-2 (y-axis) increases with the significance of P-value (x-axis) for both positive and negative effect tissue-1 eQTLs. g The directionality consistency of the shared eQTL increases with the significance of the P-value (x-axis) for both positive and negative effect tissue-1 eQTLs. [file 40104_2023_876_MOESM13_ESM.tif]

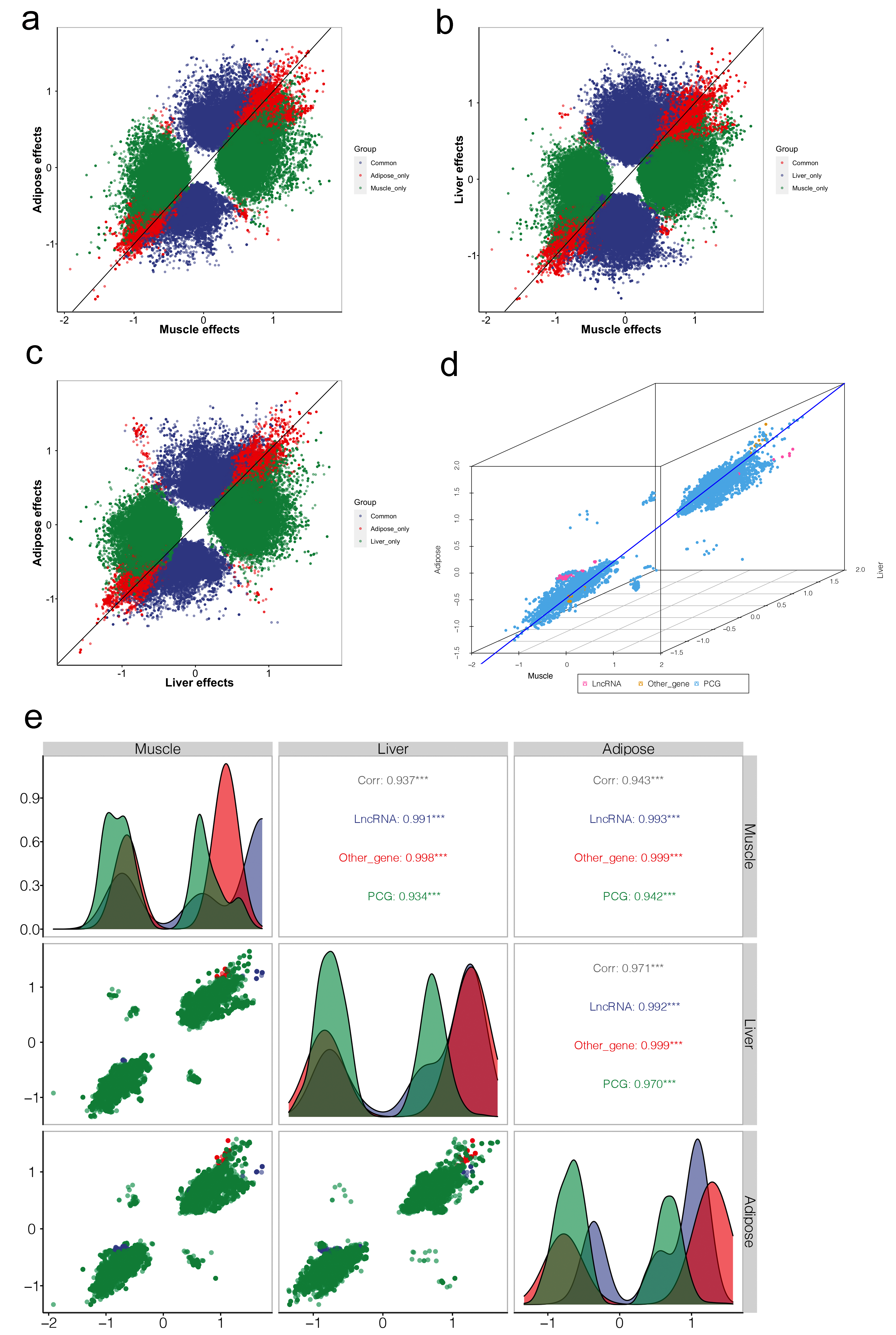

Supplement: Supplementary file 14 — Additional file 14: Fig. S14. The correlation of eQTL slope effect sizes between tissues. a Correlation between muscle and adipose. b Correlation between muscle and liver. c Correlation between liver and adipose. d Correlation of common eQTL effect sizes across all three tissues using three dimensions. e Correlation matrix plot of common eQTL effect sizes across three tissues. [file 40104_2023_876_MOESM14_ESM.tif]

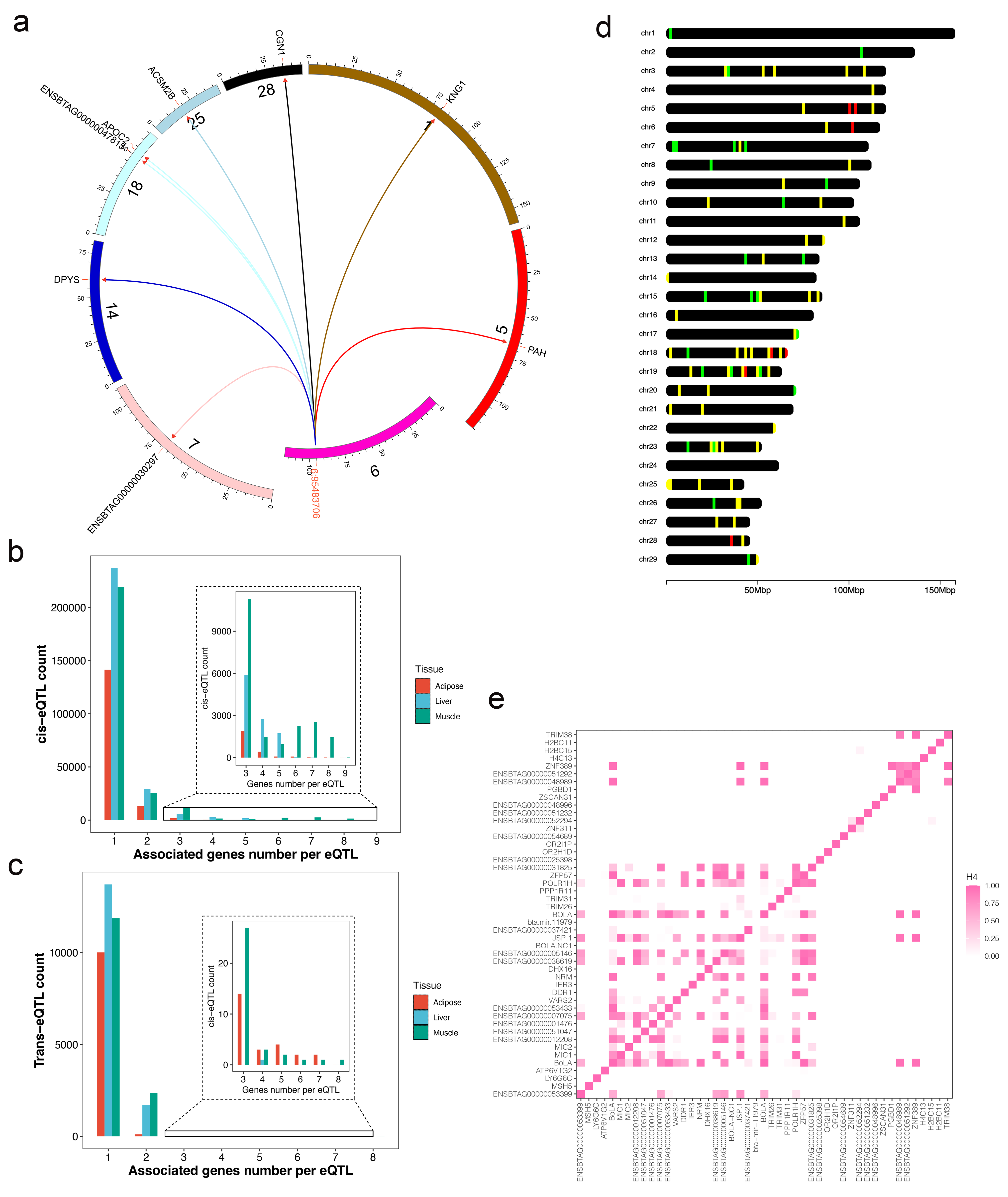

Supplement: Supplementary file 15 — Additional file 15: Fig. S15. The pleiotropic character of eQTLs. a The circos plot of the trans-eQTL pleiotropic example. b The number of cis-eGenes regulated by eVariant. c The number of trans-eGenes regulated by eVariant. d The distribution of eQTL clusters in the cattle genome. The green, yellow and red highlights represent muscle, adipose and liver, respectively. e Data showing the colocalization posterior probabilities (H4) for all 47 eGenes located in cluster 23:27612341-31472505 in adipose. H4 was colored in red with increasing intensity. [file 40104_2023_876_MOESM15_ESM.tif]

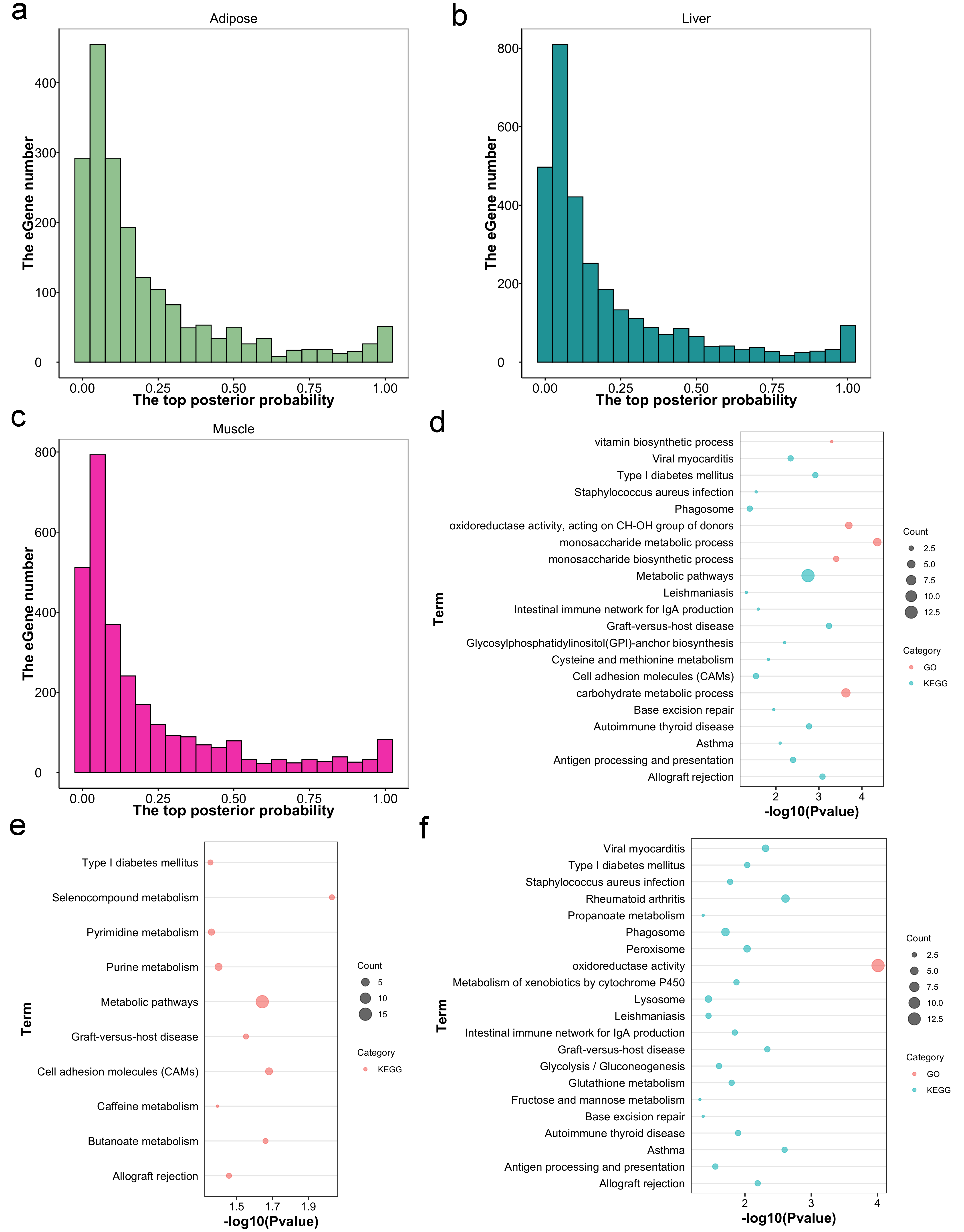

Supplement: Supplementary file 16 — Additional file 16: Fig. S16. The fine-mapping results of cis-eQTLs by DAP-g. a–c The posterior probability distribution of cis-eQTL fine mapping using DAP-g in adipose, liver, and muscle. d–f Gene enrichment for these genes with fine-mapping variants in adipose, liver, and muscle. [file 40104_2023_876_MOESM16_ESM.tif]

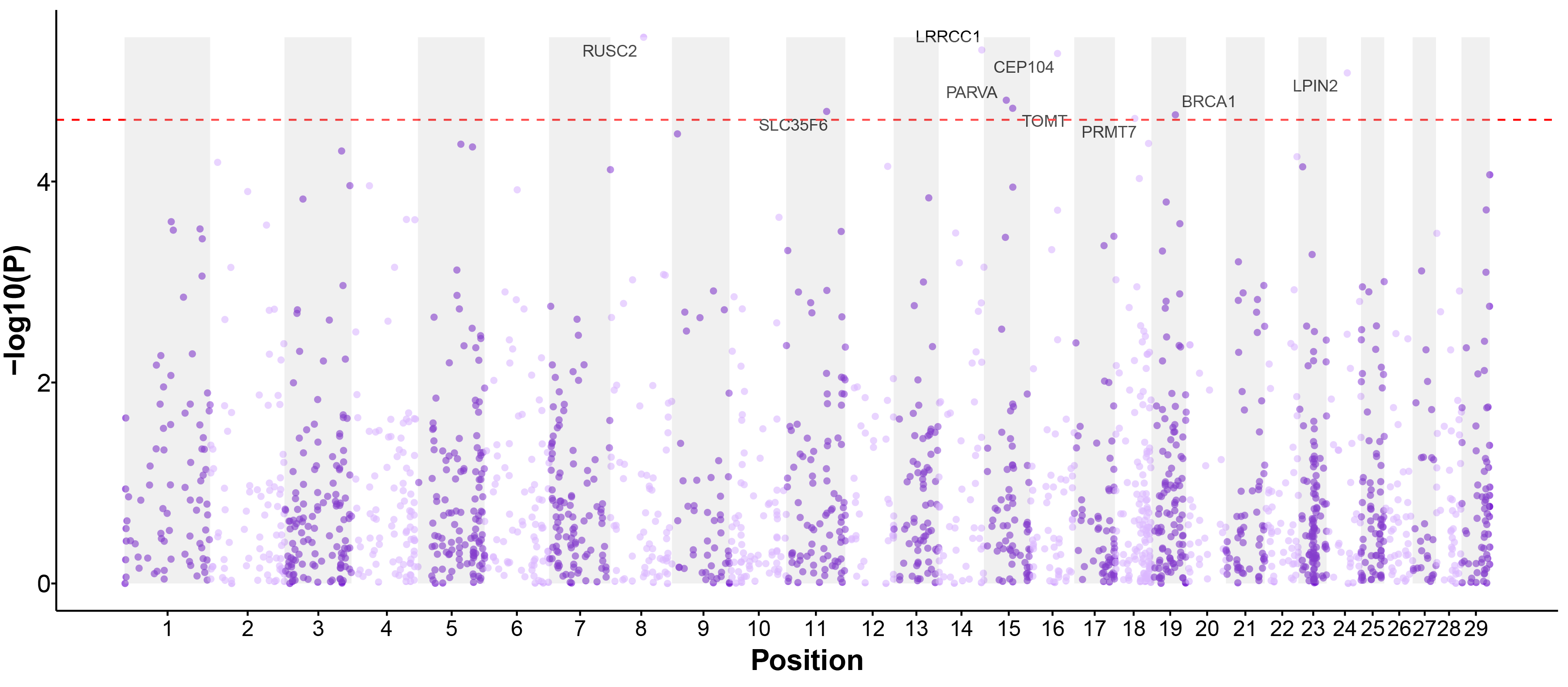

Supplement: Supplementary file 17 — Additional file 17: Fig. S17. Manhattan plot of TWAS between liver gene expression and backfat thickness. Manhattan plot showing the genomic position (x-axis) and the P-value for association (y-axis). [file 40104_2023_876_MOESM17_ESM.tif]

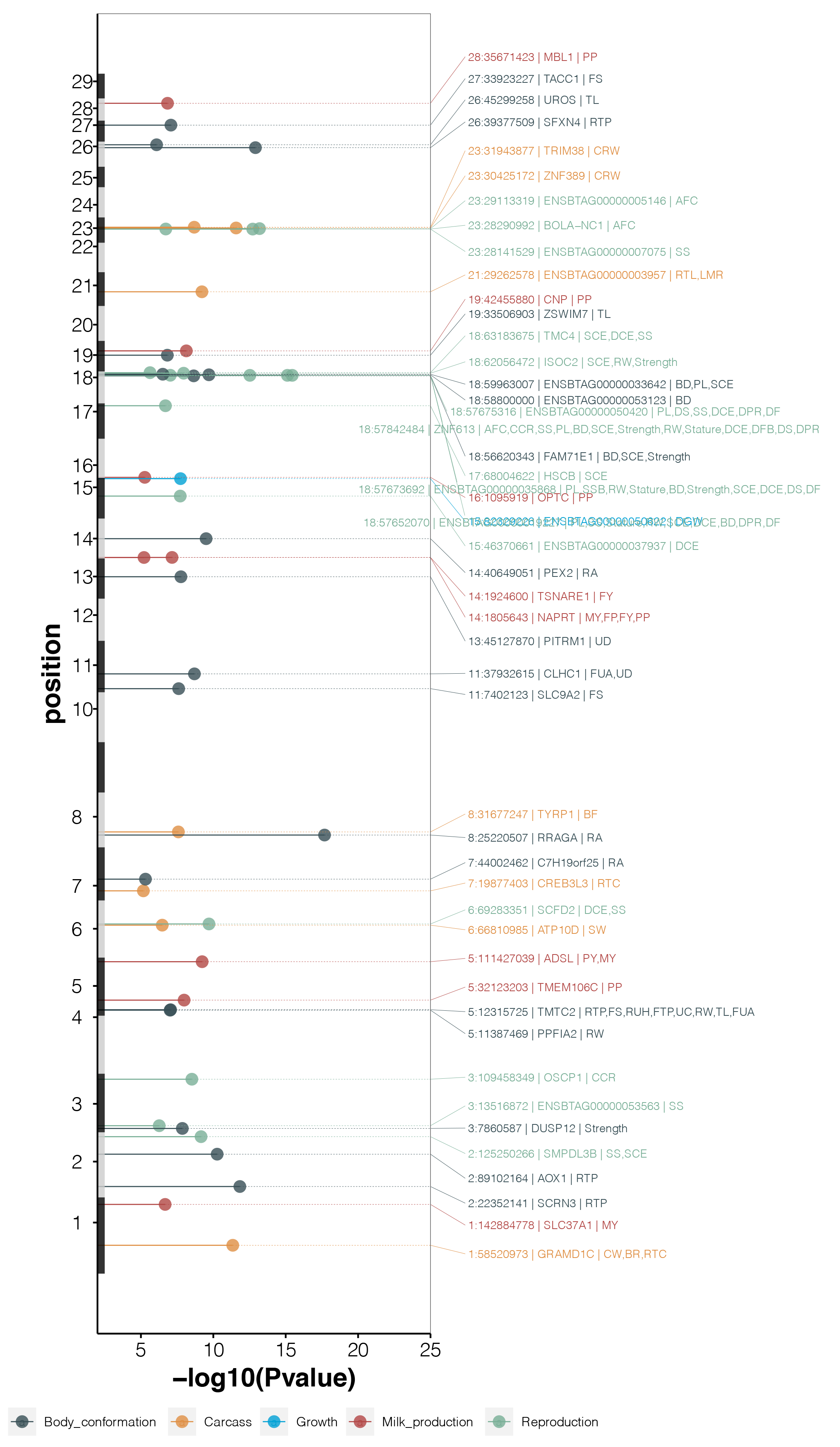

Supplement: Supplementary file 18 — Additional file 18: Fig. S18. Manhattan plot showing the colocalization results (H4 > 0.8) between eQTL and GWAS signals. The x-axis is the P-value of lead eQTLs (points) across traits (colors) in adipose. [file 40104_2023_876_MOESM18_ESM.tif]

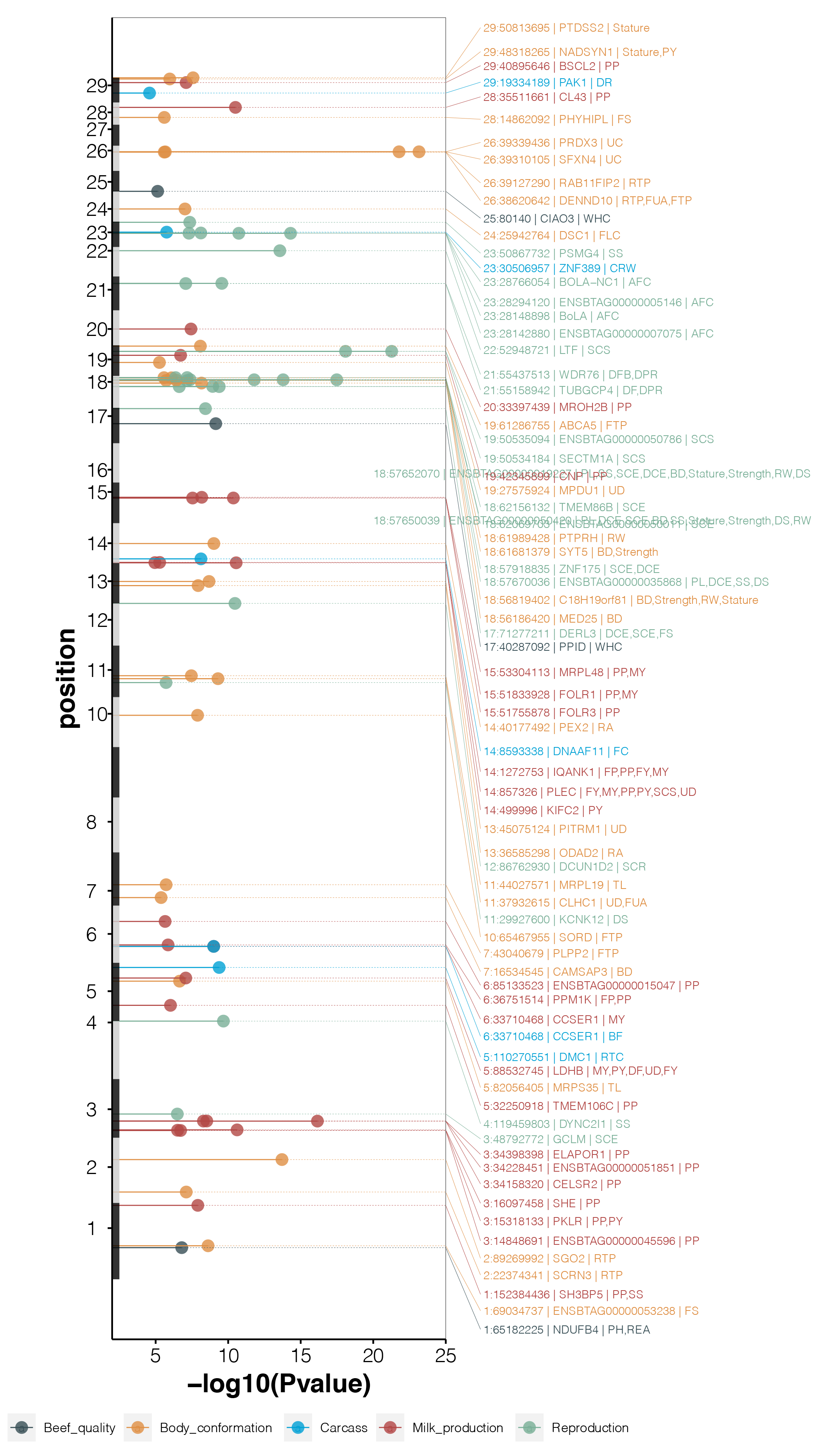

Supplement: Supplementary file 19 — Additional file 19: Fig. S19. Manhattan plot showing the colocalization results (H4 > 0.8) between eQTL and GWAS signals. The x-axis is the P-value of lead eQTLs (points) across traits (colors) in liver. [file 40104_2023_876_MOESM19_ESM.tif]

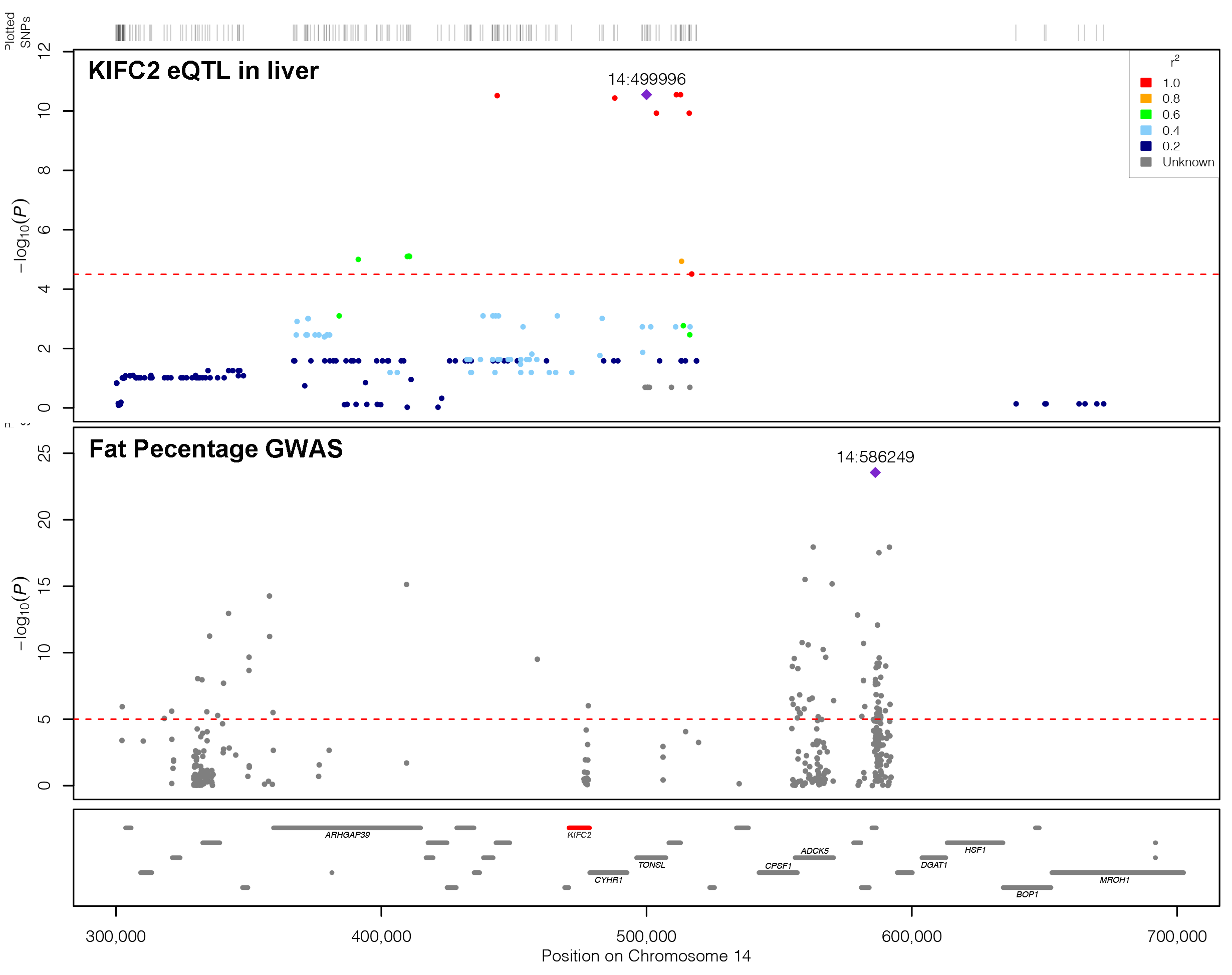

Supplement: Supplementary file 20 — Additional file 20: Fig. S20. An example of GWAS–eQTL colocalization for KIFC2 in the liver. The colors of variants are based on their LD with the most significant variant. [file 40104_2023_876_MOESM20_ESM.tif]
